# *In situ* tuning proangiogenic factor-mediated immunotolerance synergizes the tumoricidal immunity via a hypoxia-triggerable liposomal bio-nanoreactor

**DOI:** 10.7150/thno.50806

**Published:** 2020-10-25

**Authors:** Chen Chen, Shengchang Zhang, Rui Zhang, Peng Sun, Chongdeng Shi, Mohnad Abdalla, Anning Li, Jiawen Xu, Wei Du, Jing Zhang, Ying Liu, Chunwei Tang, Zhenmei Yang, Xinyi Jiang

**Affiliations:** 1 Key Laboratory of Chemical Biology (Ministry of Education), Department of Pharmaceutics, School of Pharmaceutical Sciences, Cheeloo College of Medicine, Shandong University, 44 Cultural West Road, Shandong Province 250012, PR China.; 2 Shandong University of Traditional Chinese Medicine, 4655 University Road, Shandong Province 250355, PR China.; 3 Department of Radiology, Qilu Hospital, Cheeloo College of Medicine, Shandong University, 107 Cultural West Road, Shandong Province 250012, PR China.; 4 Department of Pathology, Shandong Provincial Hospital Affiliated to Shandong First Medical University, 324 Five Weft Seven Road, Shandong Province 250021, PR China.; 5 Department of Pathology, Shandong Provincial Hospital, Cheeloo College of Medicine, Shandong University, 324 Five Weft Seven Road, Shandong Province 250021, PR China.

**Keywords:** Hypoxia-triggered liposome, Metal-polyphenol-gene bio-nanoreactor, Proangiogenic factor, Immunotolerance, Chemodynamic therapy

## Abstract

**Rationale:** Vascular abnormality stemming from the hypoxia-driven elevation of proangiogenic factors is a hallmark for many solid malignant tumors, including colorectal cancer (CRC) and its liver metastasis. We report a hypoxia-triggered liposome-supported metal-polyphenol-gene bio-nanoreactor to tune the proangiogenic factor-mediated immunotolerance and synergize the elicited tumoricidal immunity for CRC treatment.

**Methods:** With the aid of polyphenol gallic acid, Cu^2+^ ion-based intracellular bio-nanoreactor was synthesized for the delivery of small interfering RNA targeting vascular endothelial growth factor and then cloaked with a hybrid liposomal membrane that harbored a hypoxia-responsive azobenzene derivative. In hypoxic tumor, the liposomal shell disintegrated, and a shrunk-size bio-nanoreactor was burst released. Intracellularly, Cu^2+^ from the bio-nanoreactor catalyzed a Fenton-like reaction with glutathione, which efficiently converted H_2_O_2_ to •OH and enabled a chemodynamic therapy (CDT) in tumor sites. With the alleviation of proangiogenic factor-mediated immunotolerance and high production of CDT-induced tumor-associated antigens, robust tumoricidal immunity was co-stimulated.

**Results:** With colorectal tumor and its liver metastasis models, we determined the underlying mechanism of proangiogenic factor-mediated immunotolerance and highlighted that the liposomal bio-nanoreactor could create positive feedback among the critical players in the vascular endothelium and synergize the elicited tumoricidal immunity.

**Conclusion:** Our work provides an alternative strategy for exerting efficient tumoricidal immunity in the proangiogenic factor-upregulated subpopulation of CRC patients and may have a wide-ranging impact on cancer immune-anti-angiogenic complementary therapy in clinics.

## Introduction

Immunotherapy has been revolutionizing the treatment landscape of various malignancies, which enables a durable control for some of the previously incurable neoplasms, such as melanoma 1, 2. However, the majority of patients with colorectal cancer (CRC) and its liver metastasis do not derive benefit from these treatments 3, 4. Vascular abnormality is a hallmark of most solid neoplasms, which stems from the hypoxia-driven elevation of proangiogenic factors, such as vascular endothelial growth factor (VEGF) 5. Through extensive bioinformatic investigation and immunohistochemistry analysis of patients' tumor tissues, we found that the VEGF expression in CRC patients is upregulated (**Figure S1A-B**). In addition, the stratification of patients by VEGF expression shows an overall survival advantage for CRC patients with lower VEGF expression (**Figure S1C**). Clinical data indicate that over 95% of the CRC patients do not benefit from the single treatment regimen with PD-L1 antibody (atezolizumab) 6. However, when combined with a VEGF antibody, the overall response rates are greatly improved 7. In patients with unresectable hepatocellular carcinoma, atezolizumab combined with bevacizumab, a monoclonal antibody that targets VEGF, results in good overall and progression free survival outcomes 8. Thus, we hypothesized that targeting the proangiogenic factor could not only normalize the abnormal tumor vasculature but also reverse proangiogenic factor-mediated immunotolerance, which would synergize the elicited tumoricidal immunity.

**Scheme 1:**
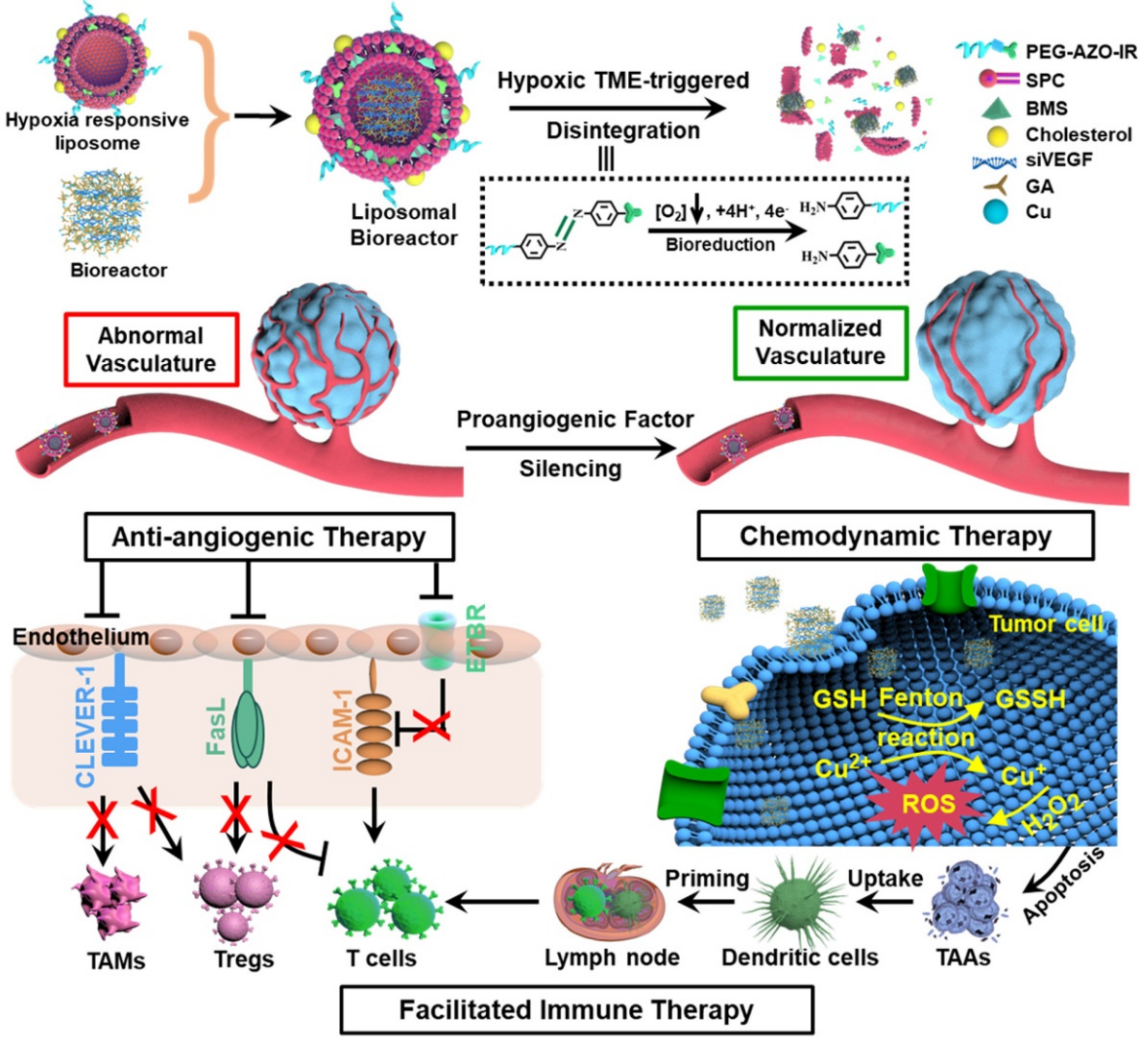
Schematic illustration that attenuating the proangiogenic factor-mediated immunotolerance with liposomal bio-nanoreactor synergized the triggered tumoricidal immunity by combination therapy.

Chemodynamic therapy (CDT), an emerging therapeutic strategy, was defined as *in situ* treatment using the Fenton reaction or Fenton-like reaction to transform intracellular hydrogen peroxide (H_2_O_2_) to reactive oxygen species (ROS, e.g., superoxide radical O_2_^•-^ and hydroxyl radical HO·) in tumor sites for triggering cell apoptosis 9-15. Considering that the Fenton reaction is substantially suppressed under slight alkaline conditions or in the presence of insufficient H_2_O_2_ in normal tissues, CDT is considered as a novel potential modality for cancer-specific treatment 16. Benefitting from the hypoxia tumor microenvironment (TME) that protected Cu^+^ from oxidation by O_2_ and consuming the antioxidant glutathione (GSH) during Fenton-like reaction, Cu^+^-catalyzation is estimated to be 160-fold higher than that of Fe^2+^ 17. High GSH-consuming efficiency breaks the delicate redox balance of cancerous cells and promotes the generation of more ROS, which is pivotal to address the issues of low efficiency in CDT 18, 19. Herein, we developed for the first time a Cu^2+^ ion-based nanocomplex for the delivery of small interfering RNA targeting VEGF (siVEGF) with the aid of polyphenol gallic acid (GA) under a facile condition (**Scheme 1**).

We designed a hypoxia-triggered liposome shell for the first time for surficial cloaking to protect the bio-nanoreactor from degradation during blood circulation. A hypoxia-responsive azobenzene (AZO) derivative, with photosensitizer IR780 (IR) as the hydrophobic part and polyethylene glycol (PEG) as the hydrophilic chain, was synthesized. Together with soybean phospholipids (SPC), PEG-AZO-IR could self-assemble into a hybrid liposome in aqueous solution. IR, as a near-infrared (NIR) fluorescent dye, could generate considerable heat under the irradiation of a NIR laser and *in situ* tumor imaging 20. Through co-extrusion of the liposomal shell and bio-nanoreactor with BMS, a PD-L1 inhibitor, the hypoxia-triggered liposome-supported metal-polyphenol-gene bio-nanoreactor (HLBBRT) was obtained. The hypoxia-triggered degradation of liposome in TME would lead to a small-size bio-nanoreactor release, which was favorable for cancer cell endocytosis. The internalized bio-nanoreactor was subsequently disintegrated with intracellular GSH, and Cu^2+^ was reduced to Cu^+^ in tumor cells, coupled with ROS generation. Cu^+^-catalyzed CDT would potently induce severe DNA damages of tumor cells. Then, the necrotic cancer cells could release tumor-associated antigens (TAAs) that could stimulate DC maturation, potentiate T cell infiltration, and exert a robust antitumor immune response 21, 22. With colorectal tumor and its liver metastasis models, we determined the underlying mechanism of proangiogenic factor-mediated immunotolerance and highlighted that the liposomal bio-nanoreactor could create positive feedback among the critical players in the vascular endothelium and synergize the elicited tumoricidal immunity by combination therapy (**Scheme 1**).

## Materials and Methods

### Preparation of BRT bioreactors

Fifteen microliters of GA (60 mM) was added into 600 μL of siVEGF (25 μM) diethylpyrocarbonate-treated water solution. Subsequently, CuCl_2_•2H_2_O (20 mM, 15 μL) was added dropwise into the abovementioned solution and stirred for 10 min to form BRT bioreactors. Then, the obtained BRT bioreactors were collected by centrifugation (5000 rpm × 5 min) and washed with water subsequently. The prepared BRT bioreactors were stored at 4 °C for the following experiments.

### Preparation of HLBBRT NPs

In brief, SPC, cholesterol, and PEG-AZO-IR with a molar ratio of 8:1:1 (w/w/w) were dissolved and then dried by a rotary evaporator to form a lipid film, which was hydrated using PBS solution. Afterward, they were extruded through a 200 nm membrane repeatedly using a hand-held liposome extruder. The same procedure was also used to prepare the liposome samples of HL, HLB, and HLBBRT on the basis of the specified compositions of the corresponding liposomes.

### Hypoxia-responsive dissolution of liposome

2.0 mg/mL of HLBBRT NPs was resuspended in 1 mL of degassed PBS containing rat liver microsomes (75 mg/mL) and 100 µM of NADPH to imitate the hypoxic TME. The morphology of HLBBRT NPs was observed using TEM images after incubation for 4 h.

### Programmed structure collapse of the BRT bioreactors

The nanoparticles, which have been incubated with hypoxic conditions, were then dispersed in 1 mL of PBS solution with 10 mM of GSH at pH 7.4 for 4 h. The morphology of the disassembled NPs after incubation was then characterized by TEM.

### Releasing profiles of BMS/siVEGF from the liposomes

HLBBRT NPs were placed in degassed PBS buffer with 0/100 µM of NADPH and 0/10 mM of GSH, with continuous shaking at 37 °C, to evaluate tumor hypoxia/GSH-responsive drug release behavior. The 200 µL of aqueous aliquots was collected at the specified time points (0, 1, 2, 4, 6, 9, 12, 24, 36, and 48 h) during the incubation period. The released media were replaced by the same fresh liquid at specific time points, and the BMS and siVEGF released from HLBBRT NPs were quantitatively measured with an HPLC spectrophotometer and microplate reader to calculate the cumulative release percentages from the complex liposomes.

### *In vitro* cellular uptake

CT26 cells were seeded in 24-well culture plates at 5×10^4^ cells/well. When the cells reached about 80% confluence, they were then incubated with different formulations containing FAM-labeled siVEGF, free IR, BRT, and HLBBRT under hypoxic conditions (1% O_2_ and 5% CO_2_) for 6 h. After rinsing with PBS, the nuclei were stained with DAPI at 4 °C in the dark for confocal laser scanning microscopy (CLSM).

### Cell cytotoxicity

For cell viability analysis, the CT26 cells were seeded into 96-well plates with a density of 5000 cells per well for 24 h and subsequently incubated with different NPs under hypoxic conditions for 12 h and then exposed to 808 nm light (1 W/cm^2^) at desired concentrations. The cells were then incubated for 24 h, followed by adding 10 µL of MTT to each well, and further cultured for 4 h. Finally, the medium was replaced with 100 µL of DMSO to dissolve formazan crystals for measuring the absorption value at 490 nm and calculating cell survival.

### *In vitro* DCFH-DA assay

ROS generation was determined through CLSM using an ROS-sensitive probe, 2′,7′-dichlorofluorescin diacetate (DCFH-DA). 5×10^4^ of CT26 cells in dishes was incubated and cultured at 5% CO_2_, 37 °C overnight, and then treated with different samples under hypoxic conditions. Subsequently, the CT26 cells were stained by DCFH-DA (10 μM) and co-incubated for 30 min. Finally, they were washed with PBS for CLSM observation.

### Orthotopic CRC animal model

4-6-week-old female BALB/c mice were anesthetized by intraperitoneal injection of pentobarbital solution. The abdomen was sterilized with alcohol swabs, and a median incision was made at the lower ventral abdomen, followed by exteriorization of the cecum. 5 × 10^5^ of CT26-luc cells in 100 µL of PBS was injected into the cecal wall. Afterward, the cecum was returned to the peritoneal cavity; the peritoneum and skin were closed with suture. Tumor formation and growth were monitored using the IVIS Spectrum (PerkinElmer, USA).

### Distribution of IR-based formulations

For tracking nanoparticle distribution in orthotopic colorectal tumor, 7 days after orthotopic tumor growth, the tumor-bearing mice (*n* = 3) were injected with IR and HLBBRT NPs through the caudal vein at an IR dose of 1 mg/kg. The *in vivo* fluorescence imaging was carried out using an IVIS imaging system at 2, 4, 8, 12, and 24 h postinjection. For *ex vivo* fluorescence imaging, the mice were sacrificed, and the major organs (i.e., heart, liver, spleen, lungs, and kidneys) and cecum were exteriorized at the end of the experiment for imaging.

### Antitumor therapy in the orthotopic colorectal tumor model

Seven days after orthotopic tumor cell inoculation, the tumor-bearing mice were treated with PBS (control), RT, siVEGF, BRT, HL, HLB, HLBRT, and HLBBRT at a siVEGF dose of 1 mg/kg and BMS dose of 4 mg/kg on days 8, 11, 14, 17, and 20. The tumor burden was monitored every 3 days using bioluminescence imaging, and the body weight was recorded throughout the study.

At the end of the study, major organs, including the intestine, heart, liver, lungs, spleen, and kidneys, were harvested for hematoxylin and eosin (H&E) histological assay to evaluate the antitumor efficacy and toxicity. In brief, the tumor tissues and major organs were fixed in 4% neutral formaldehyde, conventionally paraffin embedded, sectioned, and placed on slides. For histopathological analysis, 4 μm of the sample sections was stained with hematoxylin and eosin using a standard procedure. Furthermore, another 4 μm of tumor sections from different groups was stained for terminal transferase dUTP nick-end labeling (TUNEL) assay to analyze the cell death in tumor tissues.

### Intratumoral infiltration of immune cells

The tumor tissues were harvested and treated with 0.5 mg/mL collagenase I and 0.2 mg/mL DNAase I for 30 min to analyze the immune cells infiltrated into the tumors. After treatment by the rubber end of a syringe, the cells were passed through nylon mesh filters with a size of 70 µm and washed with cold PBS. Then, the single-cell suspension was incubated with anti-CD3-PerCP-Cy5.5, anti-CD4-FITC, and anti-CD8-PE for analysis of CD4+ T and CD8+ T cells. The single-cell suspension was stained with anti-CD4-FITC and anti-CD25-APC to analyze regulatory T cells. For MDSC analysis, the cells were labeled with anti-CD11b-APC and anti-Gr1-FITC. For M2-TAM analysis, the cells were stained with anti-F4/80 and anti-CD206 antibodies. Finally, flow cytometry and FlowJo software were used for cell data analysis.

### Antitumor therapy in CRC liver metastasis

1.5×10^5^ of CT26 cells suspended in 150 μL of PBS was injected into the distal section of the spleen via hemi-spleen inoculation to establish an animal model of CRC liver metastasis. Five minutes later, when the tumor cells entered the portal vein, the other half of the spleen was returned to the cavity. Then, the abdominal wall and skin were closed with sutures, after treatment with a diverse regime with PBS, RT, siVEGF, BRT, HL, HLB, HLBRT, and HLBBRT at the same dose as that of CRC treatment. The growth of the colon cancer liver metastasis was monitored by an IVIS system.

The quantitative methods of T cells and cytokines in sera were similar to the abovementioned methods for orthotopic colorectal tumor. In brief, livers' single-cell suspension was harvested and stained with CD4 and CD8 antibodies. Then, cells were detected by flow cytometry. Moreover, TNF-α and IFN-γ were analyzed with ELISA kits according to the protocols.

### Statistical analysis

All the data were presented as mean±S.D. Unpaired Student's t-test (two-tailed) was used for comparison between two groups. One-way analysis of variance (ANOVA) was used for multiple-group analysis. The data were expressed as mean ± SD; * *p* < 0.05, **p < 0.01, *** *p* < 0.001, and ****p < 0.0001.

## Results and Discussion

### Synthesis and characterization of liposome-supported bioreactors

**Figure 1:**
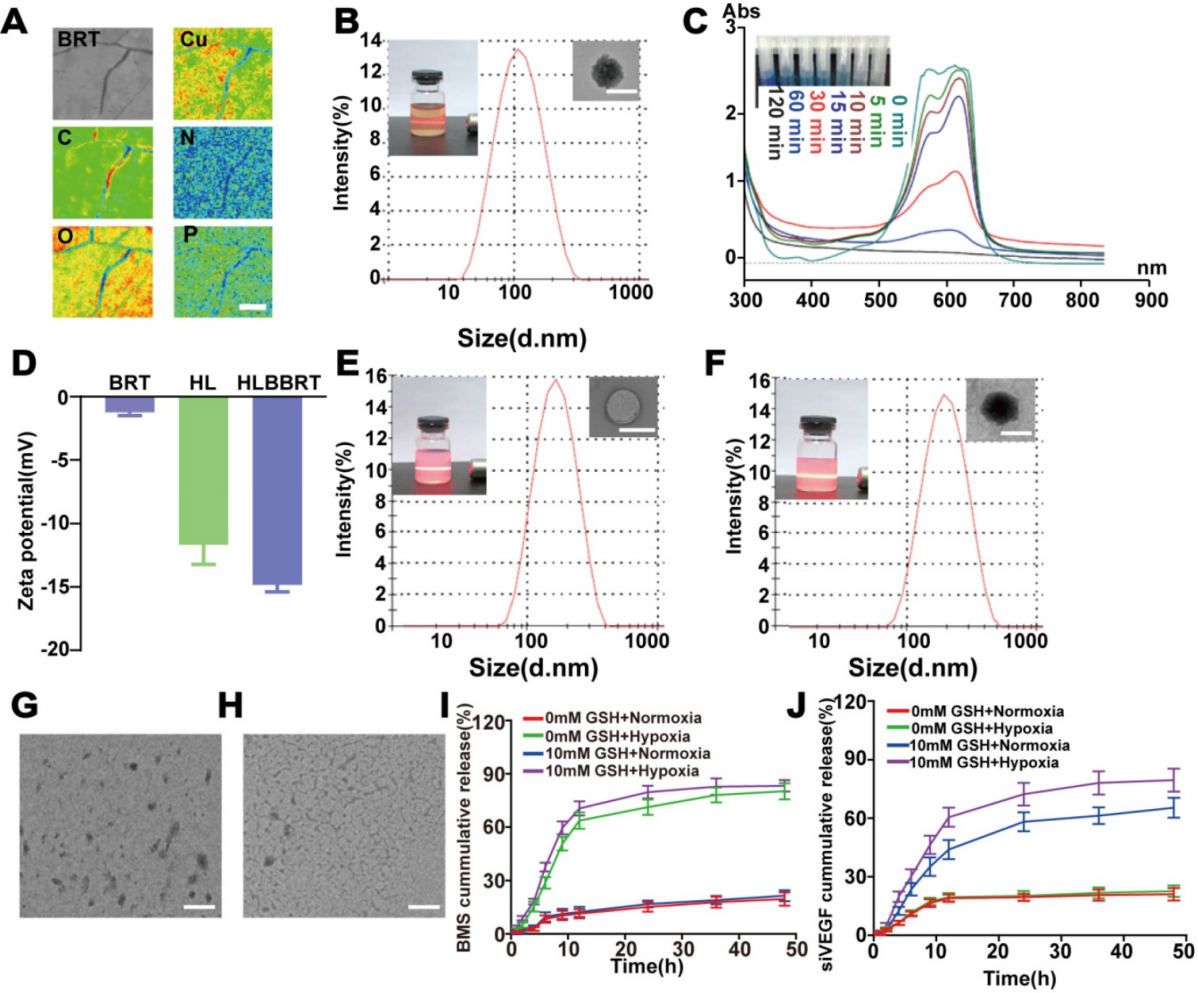
Characterization of liposome-supported bioreactors. (**A**) The primary element mapping of the BRT by EPMA (scale bar, 15 µm). (**B**) Size and morphology of the bio-nanoreactor (scale bar, 100 nm). (**C**) Time-dependent spectral change of MB degradation via BRT-initiated •OH generation. (**D**) Zeta potential of the different nanoparticles. (**E-F**) Size and morphology of the HL and HLBBRT (scale bar, 200 nm). (**G-H**) TEM images of HLBBRT that incubated under hypoxia condition without (**G**) or with GSH (**H**) (scale bar, 500 nm). (**I-J**) BMS and siVEGF release profiles from HLBBRT in different conditions.

A metal-polyphenol-gene bio-nanoreactor (BRT) was fabricated under an ultra-facile condition to develop a comprehensive nanoplatform for achieving sustainable ROS-generation with an efficient gene delivery property for tumor multimodal therapies. In brief, based on our pilot screening (**Figure S2**), the aqueous solutions of Gallic acid (GA), CuCl_2_, and siVEGF (GA vs Cu^2+^ vs siVEGF with a molar ratio of 60:20:1) were mixed under stirring. As illustrated in **Figure 1A**, the main elements were homogeneously distributed in BRT. X-ray photoelectron spectroscopy (XPS) and thermogravimetric analysis curves of Cu-GA results also confirmed the successful fabrication of a metal-polyphenol nanoreactor (**Figure S3-4**). **Figure S5** shows the XRD patterns of GA, Cu-GA, and BRT. The structural integrity of Cu-GA was unaltered after siVEGF was loaded. The bioreactor structure was then analyzed with Fourier transform infrared spectroscopy. In comparison with that of GA (**Figure S6**), the O-H stretching vibration band of GA-Cu and BRT was narrowed and shifted from 3285 to 3415 cm^-1^ and 3378 cm^-1^, indicating that Cu^2+^ was coordinated with GA moieties. Moreover, one new peak appearing at 1201 cm^-1^ indicated the successful conjugation with siVEGF. Furthermore, the dynamic light scattering assay revealed that the average diameter of BRT was 95.29 nm (**Figure 1B, Figure S7**). The •OH generation was confirmed by methylene blue (MB) solution to investigate the potential of BRT-initiated Fenton-like reaction 23. As shown in **Figure 1C**, the absorbance gradually changed in a time-dependent degradation manner, indirectly indicating the generation of •OH. Moreover, the strong ESR signals of DMPO/•OH of the BRT sample incubated with H_2_O_2_ (1 mM) demonstrated the significant production of hydroxyl radicals, whereas no signal was detected in the samples of bare BRT or H_2_O_2_ alone (**Figure S8**). These phenomena proved that the Fenton-like reaction catalyzed by BRT was efficient in the generation of highly reactive •OH.

A stealth liposomal formulation was proposed for bio-nanoreactor cloaking to increase the tumor-specific drug delivery and protect the siVEGF from enzymatic degradation in circulation. Tumor hypoxia is a notable phenomenon in TME and characteristic of limited oxygen levels inside the advanced solid tumors 24-26. Azobenzene (AZO) moiety could respond to hypoxia and be reduced to aniline derivatives 27. Given the good sensitivity of hypoxia, an amphiphilic AZO derivative was explored for the preparation of a hypoxia-responsive hybrid liposomal shell. PEG-AZO-IR was synthesized by a two-step condensation reaction. The chemical structures were confirmed through^ 1^H-NMR spectra (**Figure S9**). Together with SPC, PEG-AZO-IR could self-assemble into a hybrid liposome in aqueous solution. Through co-extrusion of the liposomal shell, the bio-nanoreactor with BMS, a PD-L1 inhibitor, the hypoxia-triggerable liposome supported metal-polyphenol-gene bio-nanoreactor (HLBBRT) was obtained. The average diameter of the blank liposome (HL) was 153.3 nm with a -11.67 mV zeta potential (**Figure 1D, E**). After co-encapsulating with BRT and BMS, the HLBBRT showed a spherical morphology under TEM imaging, with a diameter of 174.8 nm (**Figure 1F, Figure S10-S11**).

HLBBRT was incubated in simulated hypoxic condition by adding rat liver microsomes and nicotinamide adenine dinucleotide phosphate to test the hypoxia-triggered degradability 28. As shown in **Figure 1G**, HLBBRT was degraded into small pieces in a hypoxic environment because of the cleavage of the AZO linker in the liposomal shell. The liposomal bio-nanoreactor was co-incubated with GSH to determine the bio-nanoreactor-initiated Fenton-like reaction. TEM images showed that the structure of the nanoparticle completely collapsed when GSH was added (**Figure 1H**), indicating that Cu^2+^-initiated Fenton-like reaction occurred, which led to the degradation of the bio-nanoreactor. Considering the good optical adsorption of IR in the NIR region, the photothermal response of HLBBRT was also evaluated under 808 nm irradiation. Compared with BRT and BMS, the IR-containing HLBBRT showed a considerable temperature increase (**Figure S12**) and good reproducibility after three on/off laser cycles (**Figure S13**). As shown in **Figure 1I-J**, 70.56% of BMS and 60.50% of siVEGF were released from HLBBRT after incubation in 10×10^-3^ M of GSH solution under hypoxia for 12 h. By contrast, no evident drug release in normoxia condition or without GSH was detected. These results indicated a hypoxia and GSH dual-responsive drug release of the liposomal bio-nanoreactor.

### Liposomal bioreactor-mediated treatment induced a robust tumoricidal effect *in vitro*

**Figure 2:**
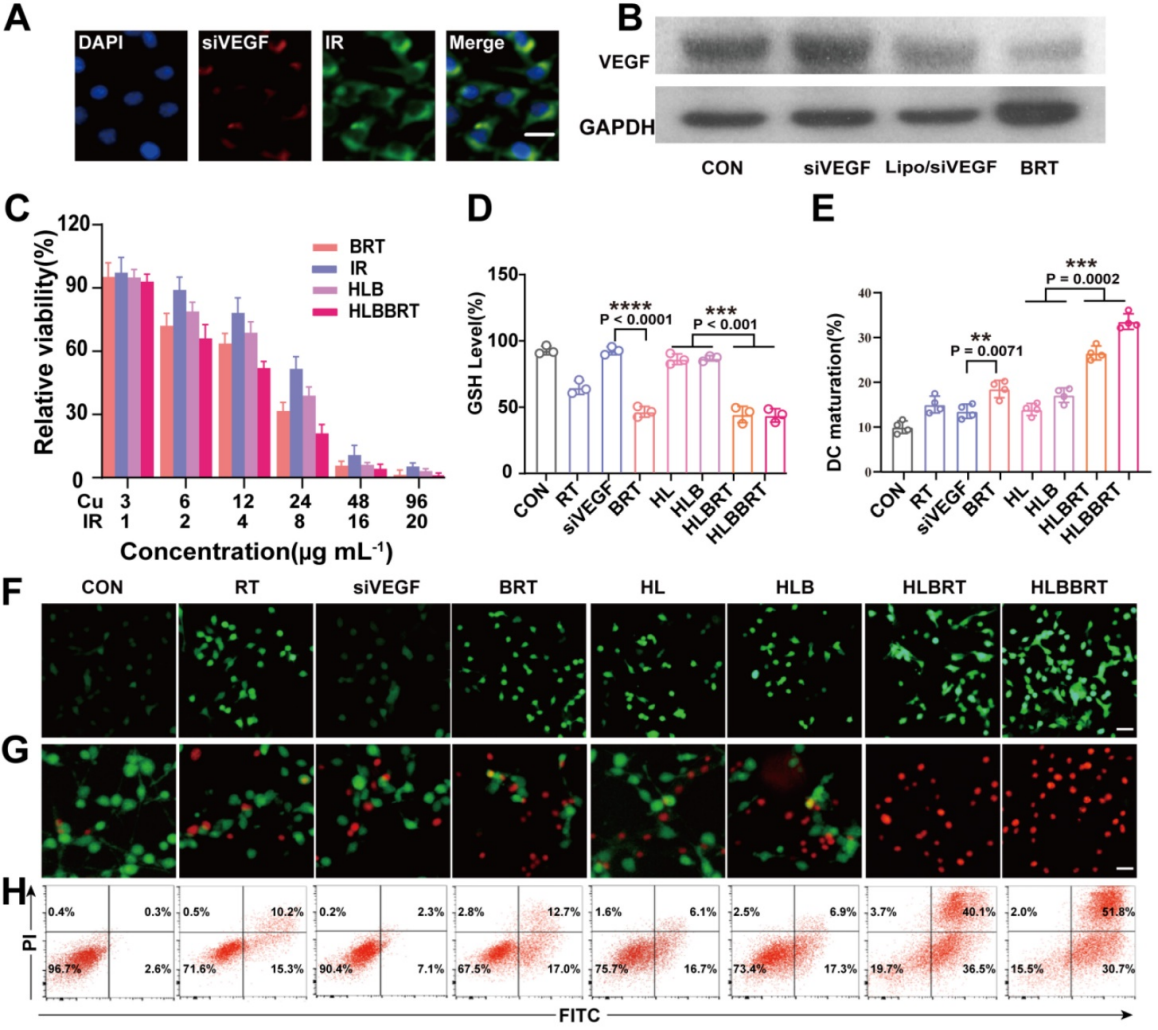
Tumoricidal effect of diverse formulations *in vitro*. (**A**) CLSM images of CT26 cells after incubation with HLBBRT (scale bar, 5 µm). (**B**) The VEGF silencing efficiency of each formulation in CT26 cells. (**C**) Cytotoxicity assay of BRT, IR, HLB, and HLBBRT against CT26 cells, *n* = 5. The full name of each group's abbreviation was shown in Table S1. (**D**) GSH level in CT26 cells treated with each formulation for 6 h, *n* = 3. (**E**) Flow cytometry assay of the DC maturation induced by each formulation,n= 4. (**F**) CLSM images of DCFH-DA-stained CT26 cells for analyzing the ROS level. Scale bar, 25 µm. (**G**) Fluorescence images of Calcein-AM and propidium iodide (PI) co-stained CT26 cells after treatment with each formulation. Scale bar, 25 µm. (**H**) Cell apoptosis after incubation with each formulation.

The successful delivery of siRNA into the cytoplasm is important for efficient gene silencing, of which cell internalization is the first step. The cellular uptake of BRT was significantly increased compared with that of the naked siVEGF as shown in CLSM (**Figure 2A, Figure S14**), indicating that siVEGF was successfully delivered into the target cells. In addition, the results of the Western blot in** Figure 2B and Figure S15** confirmed the efficient gene silencing by the BRT. Given the excellent •OH generation ability and efficient photothermal effect, HLBBRT exhibited a more potent inhibition against CT26 cells than that of BRT and HLB (**Figure 2C**). Next, we studied the Fenton-like catalytic activity of BRT with regard to Cu^2+^-mediated depletion of intracellular GSH. As shown in **Figure 2D**, the intracellular GSH dramatically decreased when incubated with BRT-mediated nanoformulations compared with that incubated with other groups, indicating that Cu^2+^ was effectively reacted with GSH, which led to an imbalance redox status in tumor cells and cell death. Furthermore, the intracellular oxidative stress after each treatment was imaged via CLSM and flow cytometry using 2′,7′-dichlorodihydrofluorescein diacetate (DCFH-DA) as the ROS probe 29. The cells treated with HLBBRT displayed the strongest green fluorescence intensity, whereas the cells treated with BRT or HLB showed the moderate one (**Figure 2F, Figure S16**). Then, live/dead cell staining assay was conducted to evaluate the antitumor effect of HLBBRT. A high red fluorescence intensity was observed in HLBBRT-treated cells, whereas a relatively low fluorescence intensity was found in either BRT or HLB-treated cells (**Figure 2G**), indicating that the optimal cytotoxicity was induced by HLBBRT and well consistent with the quantitative analysis by flow cytometry (**Figure 2H**).

Given the effect of ICD of tumor cells on priming the antitumor immune system, we evaluated whether the CDT-based antitumor therapy could trigger TAAs release and immune response. Calreticulin (CRT) protein was expressed on the cancer cell surface and served as an “eat me” signal to the antigen-presenting cells (APCs), which has been widely recognized as a biomarker of ICD effect 30-32. As shown in **Figure S17**, after treatment with RT-mediated nanoparticles, the expression of CRT exposure on the CT26 tumor cells was significantly increased under CLSM, in which redder fluorescence was observed in the group treated with HLBBRT compared with other groups. Similar results were observed *in vivo* that the stimulation of HLBBRT NPs induced the highest level of translocation of CRT on the cell membrane compared with other nanoformulations (**Figure S18**). Dendritic cells (DCs) play crucial roles in antigen presentation and T cell priming. Next, we investigated whether or not dying tumor cells induced by each formulation could activate DC maturation. Bone marrow-derived DCs were isolated from BALB/c mice and primed with the CT26 cells pre-treated with each formulation. As shown in **Figure 2E**, HLBBRT, BRT, and HLB promoted DC maturation, among which, HLBBRT showed the optimal stimulation on DCs (**Figure 2E, Figure S19**). The results indicated that high immunogenicity was induced by the HLBBRT-pretreated tumor cells, which could elicit DC maturation and imply a further immune response cycle.

### Liposomal bioreactor synergistically potentiated the inhibition of colon cancer

**Figure 3:**
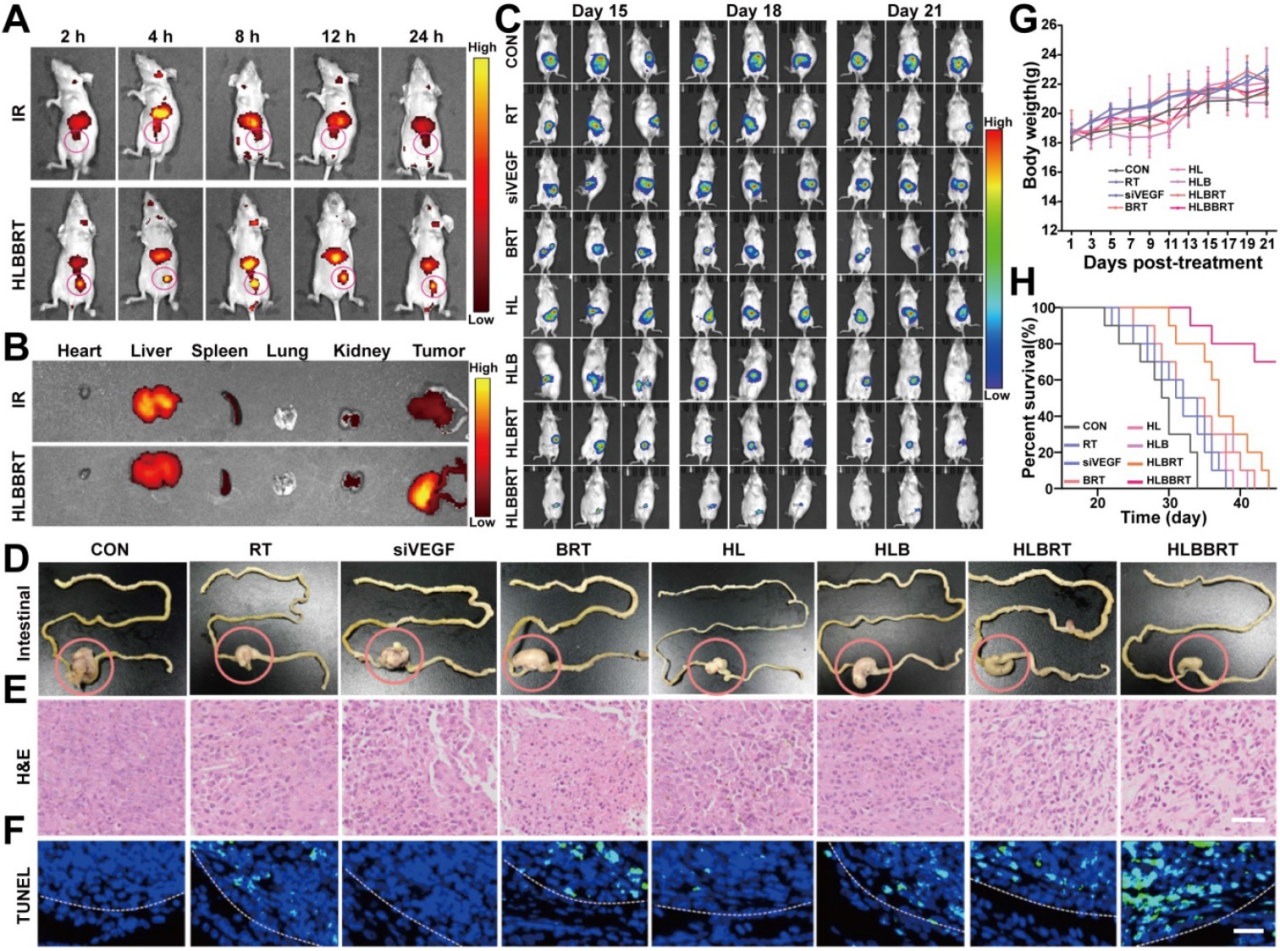
*In vivo* treatment efficacy of HLBBRT. (**A**) *In vivo* fluorescence imaging for tracking of the biodistribution of the formulations in tumor-bearing mice, *n*=3. (**B**) *Ex vivo* fluorescence imaging of major organs (heart, liver, spleen, lungs, and kidneys) and tumors harvested 24 h after intravenous injection with IR or HLBBRT, *n*=3. (**C**) *In vivo* bioluminescence imaging of mice bearing orthotopic colon cancer after diverse treatments at different time points, *n*=3. (**D**) Representative intestine tissue isolated from the mice in each treatment group at the end time point. (**E-F**) H&E (scale bar, 50 µm) and TUNEL (scale bar, 25 µm) analysis of tumor sections from the treated mice. (**G**) Body weight changes of mice in each group. (**H**) Survival curves of mice in different groups.

By IVIS imaging system, we determined the distribution of the formulations in colon cancer-bearing mice. As shown in **Figure 3A**, the fluorescence intensity of the IR in HLBBRT was remarkably stronger than that of the free IR at the corresponding time. The fluorescence signal increased with the increase of time, indicating that HLBBRT was accumulated and retained in tumors continuously. The preferential tumor accumulation was reconfirmed by the *ex vivo* fluorescence imaging, which was harvested at 24 h (**Figure 3B**). Next, the antitumor activity of HLBBRT was assessed *in vivo*. An orthotopic CRC model was established by injection of murine CT26 colon cancer cells into the cecum wall of BALB/c mice 33. As shown in** Figure 3C**, BRT and HLB showed minimal tumor growth inhibition. The combined therapy with HLBBRT induced the premier tumor inhibition. Twenty-one days postinjection, the representative photographs of the intestine showed that HLBBRT achieved a superior therapeutic effect compared with other treatments (**Figure 3D**). Images from both H&E staining and TUNEL analysis showed that the tumor sections in the HLBBRT-treated group exhibited severe necrosis and apoptosis (**Figure 3E-F**), which was consistent with the tumor inhibition efficacy. In addition, we investigated the oxidative stress of tumor samples in each group. **Figure S20** shows that HLBBRT NPs exhibited the strongest DCF fluorescence signals. By contrast, GSH consumption in tumors of the HLBBRT NP treatment group was higher than that of other treatment groups (**Figure S21**). These data indicated that HLBBRT NPs had greater tumor inhibition through antioxidant defenses via elevated highly toxic ROS levels and GSH depletion. Moreover, histopathological evaluation (**Figure S22**) revealed that no visible damage of the main organs was induced in mice from all treatment groups, indicating an efficient biocompatibility of the nanoformulations. Synergizing the cascade cancer multimodal therapies, the liposome-supported bio-nanoreactor significantly improved the survival of the colon cancer-bearing mice without decreasing the body weight (**Figure 3G-H**).

### VEGF silencing with liposomal bioreactor attenuated tumor-immune inhibition and created an activated tumoricidal immunity

**Figure 4:**
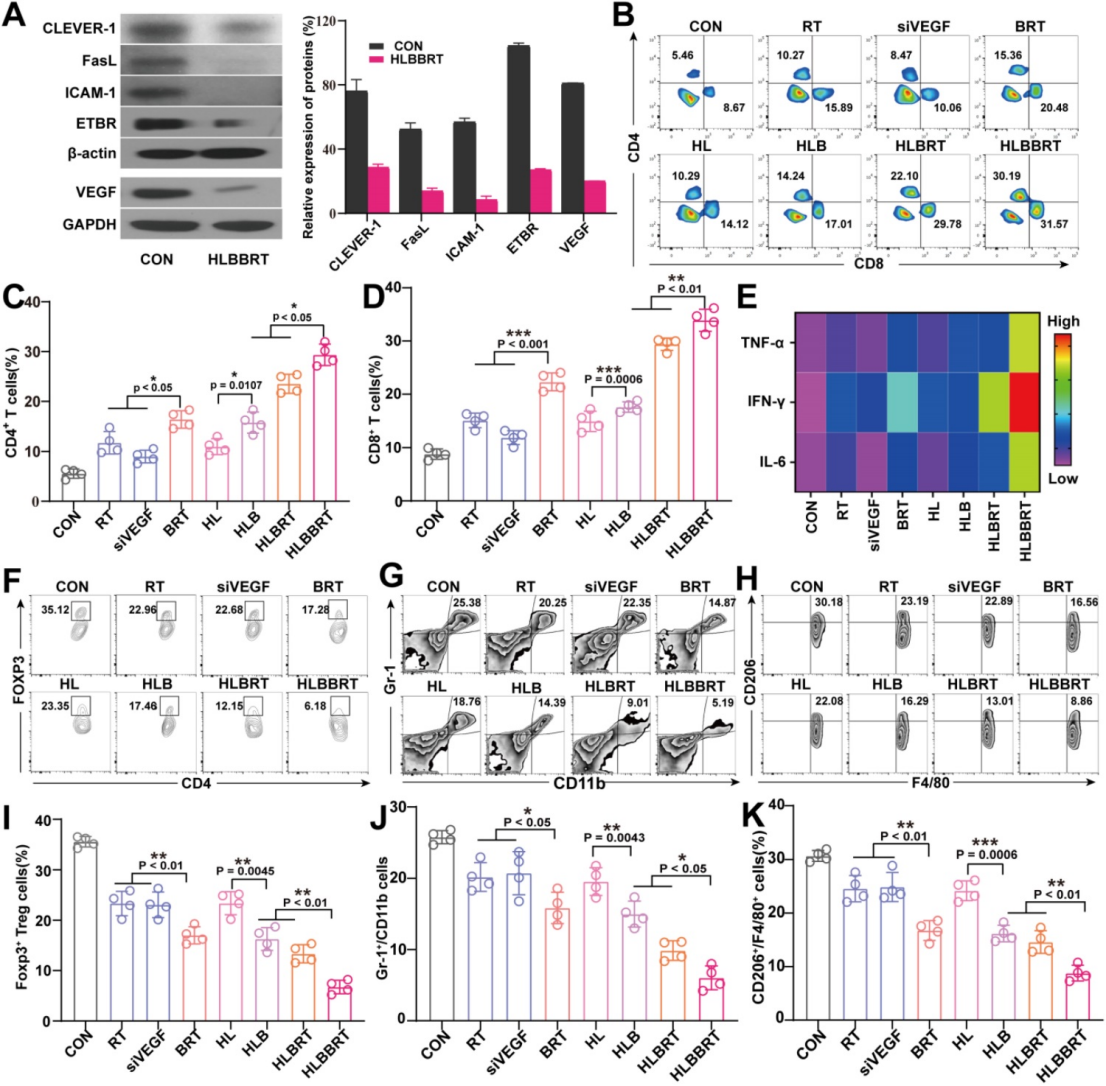
Tumoricidal immunity triggered by the liposomal bioreactor-mediated multimodal treatments. (**A**) The analysis of protein expression after treatment with HLBBRT. (**B-D**) Representative flow cytometry and quantitative analysis of the CD3^+^/CD4^+^ and CD3^+^/CD8^+^ T cells. (**E**) The heat map of the major cytokines in the sera of mice. (**F-H**) Flow cytometry analysis of the CD4^+^/Foxp3^+^ Tregs (**F**), CD11b^+^/Gr1^+^ MDSCs (**G**), and F4/80^+^/CD206^+^ M2-TAM (**H**). (**I-K**) Quantitative analyses of the Tregs (**I**), MDSCs (**J**), and M2-TAM (**K**), *n* = 4.

The expression of VEGF was evaluated in mice after different treatments to demonstrate the anti-angiogenic effect of the diverse formulations. As evidenced by the faintly visible protein bands (**Figure 4A**), the expression of VEGF following intravenous administration could be effectively downregulated in the HLBBRT group. Consistent with the results of VEGF gene silencing, the HLBBRT also exerted the most effective *in vivo* cell adhesion molecule downregulation, involving endothelin B receptor (ETBR) through modulation of the endothelium via the upregulation of intercellular adhesion molecule-1 (ICAM-1), Fas ligand (FasL), and common lymphatic endothelial and vascular endothelial receptor-1 (CLEVER-1), which were mediated by VEGF expression and in turn stimulated immunosuppressive TME. Moreover, the expression of HIF-1α and angiogenic markers CD31 was estimated. As shown in **Figure S23** and **Figure S24**, compared with the control group, tumors from mice injected with HLBBRT NPs showed significantly lower expression. These results indicated that HLBBRT nanoparticle treatment resulted in a significant decrease in tumor vascularization and low expression of HIF-1α protein because of the combination of cytotoxic and anti-angiogenic activity in a synergistic nanosystem. Based on previous reports, orthotopic colorectal tumor harbored a stringent immune suppressive TME 34. Tumors from the diverse treatment groups were harvested to determine if tumor-infiltrating immune cells in the tumor site could be appropriately activated, and a single cell suspension was prepared. After immune staining of each biomarker, the cells were analyzed by flow cytometry. As displayed in **Figure 4B-D**, the HLBBRT treatment led to a remarkable infiltration of CD4^+^ and CD8^+^ T cells, and quantitatively, the percentage of CD8^+^ T cells was approximately 1.5-fold higher than that in the BRT or HLB-treated groups. The significant inhibition of tumors in the HLBBRT-treated group might be attributed to the increased infiltration of CD8^+^ T in tumor tissues. Furthermore, the highest level of cytokines, including TNF-α, IFN-γ, and IL-6, was determined in sera from the HLBBRT group compared with other groups, indicating that the liposomal bio-nanoreactor activated the innate antitumor immune response 35 (**Figure 4E**). Excessive levels of proangiogenic factors, such as VEGF in the TME, induced immunosuppression, which aborted the antitumor immunity. Silencing proangiogenic factors could reduce the immunosuppressive cell populations. As shown in **Figure 4F-H**, a significant reduction of the immune suppressor, including Tregs, MDSCs, and M2-TAM, was observed in the siVEGF-based nanoformulation group. The content of Tregs in tumor, which was correlated with the poor prognosis of cancer patients 36, was reduced by 81.0% in HLBBRT treated mice compared with that of the controls (**Figure 4I**). We analyzed the ratios of effector T cells (CD8+/CD4+ T cells) to Tregs to investigate the synergistic effects of our prepared HLBBRT formulation for enhanced tumor infiltration of these immune cells. The results in **Figure S25** indicated that the ratios of CD8+ T cells and CD4+ T cells to Tregs increased remarkably, in accordance with the reduced proportions of tumor-protective Tregs after a series of antitumor therapy (**Figure 4F**), demonstrating the positive influence of the synergistic therapy based on the HLBBRT formulation on promoting systemic immune response. In addition, in the PBS group, the percentage of MDSCs was 25.8% ± 0.9%, which decreased to 6.0% ± 1.7% in HLBBRT-treated mice (**Figure 4J**), all of which implied an attenuated immuno-resistance in TME upon treatment with HLBBRT. Through immune suppression, M2-TAM promoted tumor development in primary colon cancer and its metastatic tumors 37. After treatment with the combination regimen HLBBRT, M2-TAM was significantly inhibited (**Figure 4K**). The abovementioned results indicated that the combined liposomal bio-nanoreactor spatiotemporally modulated the immune suppressive TME and converted the ICB nonresponding tumors into responding ones by increasing the infiltration of immune effector cells while reducing the frequency of immunosuppressive cells in tumors, thereby inducing multiple impacts on tumoricidal immunity.

### Treatment with liposomal bioreactor eradicated the colon to liver metastasis

**Figure 5:**
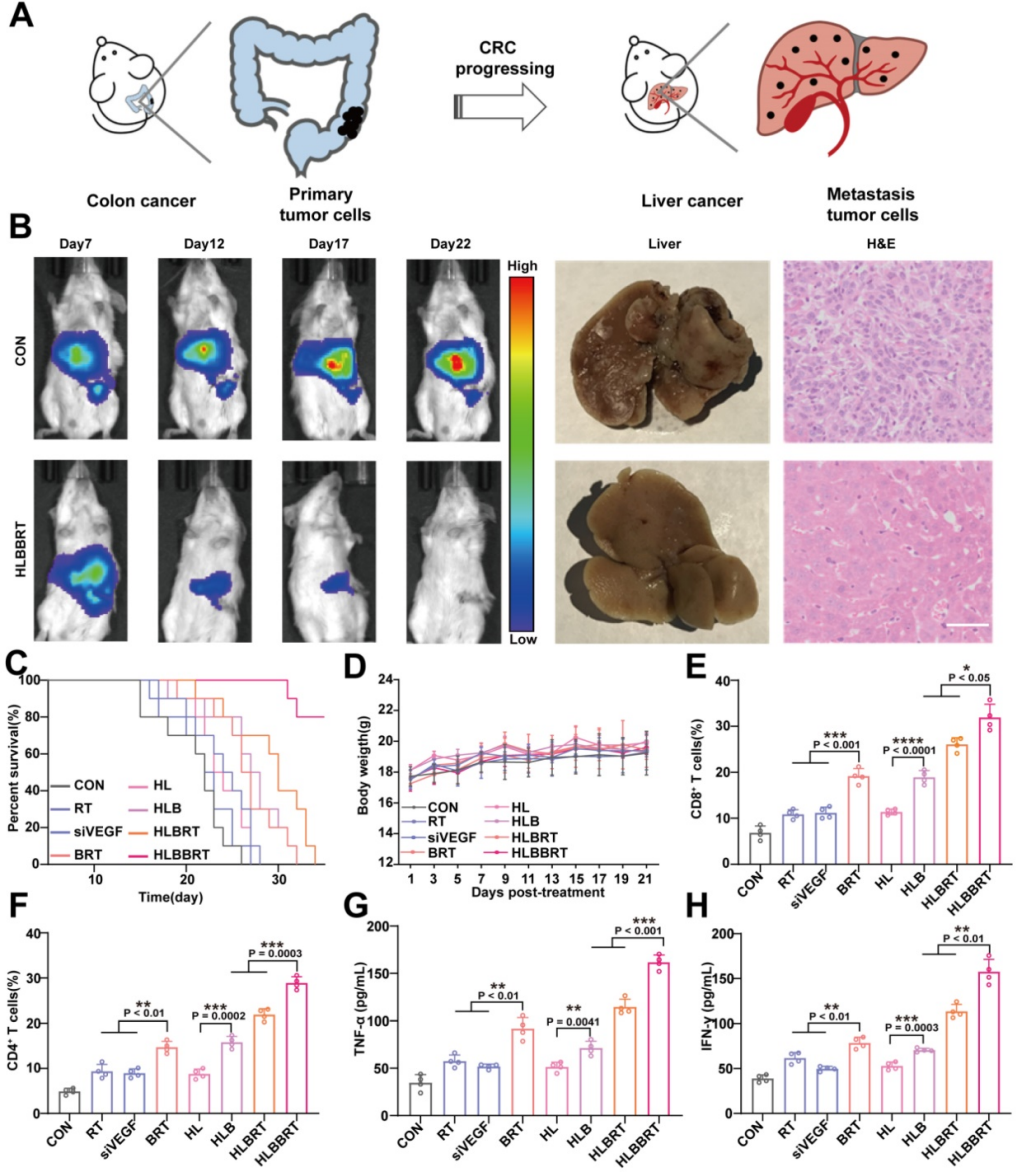
*In vivo* antitumor evaluation of HLBBRT in colon cancer liver metastasis. (**A**) Schematic diagram demonstrating primary colon cancer and its liver metastasis. (**B**) Bioluminescence imaging for tracking the tumor growth and representative photographs and H&E (scale bar, 50 µm) of the isolated liver. (**C**) The survival curves of mice, *n*=10. (**D**) The body weight changes of mice in each group. (**E-F**) Quantification of the CD8^+^ and CD4^+^ T cells after each treatment. (**G-H**) Cytokine-secreting levels in each treated group.

The liver is the primary metastatic site for CRC (**Figure 5A**), a significant cause of CRC-related death 38. We established a liver metastasis model by hemi-splenic inoculation of CT26 cells to evaluate the potency of our combined therapy on countering CRC liver metastasis 38. As shown in **Figure 5B and Figure S26**, CT26 tumor cell growth in the liver was significantly inhibited after treatment with HLBBRT compared with that of the control groups, and antitumor trend was achieved by liver imaging and H&E staining analysis (**Figure 5B, Figure S27**). In addition, the survival of the mice in the HLBBRT group was significantly prolonged (**Figure 5C**), and no significant difference in body weight was detected among the treated groups (**Figure 5D**). With regard to the mechanism, CD8^+^ and CD4^+^ T cells in the HLBBRT group significantly increased up to 4.6- and 5.9-fold compared with the control groups (**Figure 5E-F, Figure S28**). Moreover, consistent with the T cell population profile, an upregulated production of proinflammatory cytokines, including TNF-α and IFN-γ, was observed after treatment with HLBBRT, indicating that a robust tumoricidal immunity was triggered by combined therapy (**Figure 5G-H**).

## Conclusion

We report a hypoxia-triggered liposomal metal-polyphenol-gene bio-nanoreactor for *in situ* tuning proangiogenic factor-mediated immunotolerance and synergizing the tumoricidal immunity elicited by CDT. The liposomal shell revealed a hypoxia-dependent disintegration both *in vitro* and *in vivo*. Intracellularly, Cu^2+^ from the bioreactor catalyzed a Fenton-like reaction with glutathione, which efficiently converted H_2_O_2_ to •OH and enabled a CDT in tumor sites. The alleviation of proangiogenic factor-mediated immunotolerance and increased production of TAA_S_ co-stimulated robust tumoricidal immunity. Mechanistic studies demonstrated that anti-angiogenic therapy alleviated proangiogenic factor-mediated immunotolerance through (i) blocking the usual upregulation of ETBR and subsequently promoting the clustering of ICAM-1, which selectively increased lymphocyte adhesion and extravasation, (ii) inhibiting CLEVER-1 and attenuating the infiltration of Tregs and M2-TAM, and (iii) downregulating the expression of endothelial FasL and synergistically decreasing the enrichment of Tregs while relieving the inhibition of cytotoxic lymphocytes in the TME. With colorectal tumor and its liver metastasis models, we highlighted that tuning proangiogenic factor-mediated immunotolerance with the hypoxia-triggered liposomal metal-polyphenol-gene bio-nanoreactor could create positive feedback among the critical players in the vascular endothelium and synergize the tumoricidal immunity elicited by CDT. Our work provided an alternative strategy for exerting efficient tumoricidal immunity in the proangiogenic factor-upregulated subpopulation of CRC patients and might obtain a wide-ranging impact on cancer immune-anti-angiogenic complementary therapy in clinics.

## Supplementary Material

Supplementary figures and tables.
