# Supplementary material for: In situ tuning proangiogenic factor-mediated immunotolerance synergizes the tumoricidal immunity via a hypoxia-triggerable liposomal bio-nanoreactor
Source: Theranostics. 2020 Oct 25;10(26):11998–2010. doi: 10.7150/thno.50806 (PMC7667679; doi:10.7150/thno.50806)
Supplement: Supplementary file 1 — Supplementary figures and tables. [file thnov10p11998s1.pdf]

Supplementary material

In situ tuning proangiogenic factor-mediated immunotolerance synergizes the tumoricidal immunity via a hypoxia-triggerable liposomal bio-nanoreactor

Chen Chen[1], Shengchang Zhang[1], Rui Zhang[1], Peng Sun[2], Chongdeng Shi[1], Mohnad Abdalla[1], Anning Li[3], Jiawen Xu[4,5], Wei Du[1], Jing Zhang[1], Ying Liu[1], Chunwei Tang[1], Zhenmei Yang[1], Xinyi Jiang[1*]

[1]Key Laboratory of Chemical Biology (Ministry of Education), Department of Pharmaceutics, School of Pharmaceutical Sciences, Cheeloo College of Medicine, Shandong University, 44 Cultural West Road, Shandong Province 250012, PR China

[2]Shandong University of Traditional Chinese Medicine, 4655 University Road, Shandong Province 250355, PR China

[3]Department of Radiology, Qilu Hospital, Cheeloo College of Medicine, Shandong University, 107 Cultural West Road, Shandong Province 250012, PR China

[4]Department of Pathology, Shandong Provincial Hospital Affiliated to Shandong First Medical University, 324 Five Weft Seven Road, Shandong Province 250021, PR China

[5]Department of Pathology, Shandong Provincial Hospital, Cheeloo College of Medicine, Shandong University, 324 Five Weft Seven Road, Shandong Province 250021, PR China

* Corresponding author:

Xinyi Jiang, Ph.D., Professor

Phone/Fax：+86-0531-88382015

Email：xinyijiang@sdu.edu.cn

Experimental section

Materials

BMS (BMS-1166, with Cat. No. of HY-102011), $CuCl_2{\cdot}2H_2O$, Gallic acid, and mPEG-NH_2 were obtained from Shanghai Macklin Biochemical Co., Ltd. IR780 was purchased from Tianjin Xi'an Sipude Technology Co., Ltd. SPC and cholesterol were provided by Shanghai Aiweite Pharmaceutical Technology Co., Ltd. 5,5′-Dithiobis (2-nitrobenzoic acid; DNTB) and MTT were all purchased from Dalian Meilun Biotech Co., Ltd. 2′,7′-Dichlorofluorescein diacetate (DCFH-DA), calcein AM, propidium iodide (PI), and annexin V-FITC were purchased from Solarbio Life Sciences. Antibodies were purchased from Bio Legend, Inc. The siRNA targeted to vascular endothelial growth factor (VEGF) mRNA (siVEGF) was purchased from Tsing Ke biological Technology. The chemicals and reagents used in this study were of analytical grade.

Cell lines and animals

CT26 colorectal cancer cells were obtained from the cell bank of the Chinese Academy of Sciences (Shanghai, China).

BALB/c and nude mice were purchased from Vital River Laboratory (Beijing, China). All animal experiments were carried out following the guidelines evaluated and approved by the ethics committee of Shandong University Animal Experiment Ethics Review and Health Guide for the Care and Use of Laboratory Animals of National Institutes.

Synthesis of IR-NH2

In brief, 0.6 mM of IR and 3 mM of 3-amino-1-propanol were dissolved in 20 mL of anhydrous DMF, followed by the addition of 170 μL of triethylamine. The reaction system was stirred at 85 °C for 6 h in the dark. The crude product was purified through a silica gel column

with the gradient elution of methanol and dichloromethane (DCM).

Synthesis of PEG-AZO

PEG (150 mg, 0.075 mM) and AZO (75 mg, 0.375 mM) were dissolved in DCM and trichloromethane (TCM) (V_{DCM}:V_{TCM}=1:1), and then, DMAP (11 mg, 0.09 mM) and DCC (15.5 mg, 0.075 mM) were added. The reaction was stirred at room temperature (RT) for 24 h. The product was obtained by precipitating three times in cold diethylether.

Synthesis of PEG-AZO-IR

The method of synthesizing PEG-AZO-IR was similar to the abovementioned procedure with a minor modification. The DCM and TCM mixed solution containing 0.02 mM of PEG-AZO and 0.02 mM of IR-NH2 was added with 0.024 mM of DMAP and 0.02 mM of DCC. Then, the solution was continuously stirred for 24 h at RT. The product was then processed with a rotary evaporator and precipitated with cold diethylether. The successful synthesis of PEG-AZO-IR was confirmed by ^{1}H-NMR.

Photothermal effect induced temperature change of different formulations

The photothermal effect of the different formulations was evaluated by recording the increasing temperature of the corresponding aqueous solutions (10 μg/mL, IR) under the irradiation of an 808 nm laser at 1 W/cm^2 using an IR thermal camera. Three photothermal cyclings of laser on/off experiment were performed to investigate the photothermal conversion stability of the IR-based HLBBRT NPs. In each cycle, the solution of the HLBBRT was exposed to an 808 nm laser and then naturally cooled down to room temperature without laser exposure.

Western blotting

CT26 cells were seeded on a 6-well plate and cultured at 5% CO_2 and 37 °C overnight. Then,

they were treated with siVEGF, LiposiVEGF, and BRT at a siVEGF dose of 50 nM for 48 h. The cells were washed with precooled PBS three times and lysed in RIPA buffer supplemented with PMSF. The protein samples were separated by 15% SDS-PAGE gel electrophoresis, transferred to PVDF membranes, followed by blocking in TBST containing 5% skim milk, and incubated with the indicated antibodies and secondary antibodies.

Endothelial cells were isolated and collected through collagenase digestion and then incubated in red blood cell lysis buffer. Subsequently, the endothelial cells were isolated by immunomagnetic selection using Abs against CD31 conjugated to Dynabeads. The expression of ETBR, ICAM-1, CLEVER-1, and FasL was analyzed by Western blot according to the abovementioned methods.

Intracellular GSH measurement

Colon cancer CT26 cells were seeded on a 6-well cell plate with the density of 1×10^6/well and cultured at 5% CO_2 and 37 °C overnight. Then, they were treated with PBS, RT, siVEGF, BRT, HL, HLB, and HLBBRT under hypoxic conditions for 6 h. The dosages for nanoformulations and siVEGF were 20 μg/mL and 50 nM, respectively. Afterward, the cells were harvested and washed with cold PBS three times and lysed with Triton-X-100 lysis buffer. After centrifugation, DTNB solution was added into the lysis buffer for 30 min coincubation, and the content of cellular GSH was determined via UV–vis absorbance at 412 nm.

Expression of CRT in vitro and in vivo

CT26 tumor cells were cultured in a 24-well plate overnight and then treated with PBS, RT, BRT, HL, or HLBBRT at a dosage of 20 μg/mL for 6 h. Afterward, the cells were washed three times with cold PBS, fixed with 4% paraformaldehyde, and incubated with Alexa fluor 647-CRT for 90 min. Finally, the cells were stained with DAPI and observed by CLSM.

The colon tumor tissues were harvested and fixed with 4% paraformaldehyde for 48 h and then subjected to sucrose gradient dehydration for the preparation of the cryosection slices. The tissue sections were then stained with CRT and DAPI for imaging by confocal laser scanning microscopy.

Maturation of dendritic cells

Bone marrow-derived dendritic cells (BMDCs) were generated from the bone marrow of 8 week-old BALB/c mice. In brief, the bone marrow cells were seeded in a 6-well plate in medium containing GM-CSF (20 ng/mL) and IL-4 (10 ng/mL) for 8 days to be differentiated into BMDCs. Then, 5×10^5/well of BMDCs and 8×10^4/well of CT26 cells were seeded into the culture plates and transwell for incubation overnight. Subsequently, fresh medium containing PBS, RT, siVEGF, BRT, HL, HLB, HLBRT, and HLBBRT (the dosages for nanoformulations and siVEGF were 20 μg/mL and 50 nM, respectively) was added into the transwell for 6 h treatment of CT26 cells. Afterward, the transwells with residues of CT26 cells were moved to the BMDC-seeded 24-well plates for 24 h co-incubation. Finally, the BMDCs were collected and washed two times with PBS, followed by staining with anti-CD11c-FITC, anti-CD86-PE, and anti-CD80-APC antibodies. The maturation of DC cells was investigated by using flow cytometry.

Cell apoptosis and necrosis analysis

Calcein-AM and PI staining of living and dead cells were conducted to visualize the cell-killing efficacy. 5×10^4 CT26 cells were incubated onto a cell plate and cultured overnight. Then, the cell medium was replaced with fresh medium containing different nanoparticles with dosages of 20 μg/mL and 50 nM for nanoformulations and siVEGF, respectively. After treatment under hypoxic conditions for 24 h, the cells were stained by calcein-AM (1 μg/mL) and PI (1 μg/mL) for 30 min at 37 °C and washed with PBS several times. The cells were

monitored by CLSM.

For cell apoptosis analysis, after incubation in 12-well plates overnight, the CT26 cells were then treated with different nanoparticles with the same dosage as that of the living and dead cell assay under hypoxic conditions for 24 h. Afterward, the cells were preincubated with 5 μL of annexin V in the dark at RT for 15 min, followed by the addition of 10 μL PI. After staining, they were washed with cold PBS three times and analyzed by flow cytometry.

In vivo ROS detection and GSH assessment

The tumor-bearing mice were randomly divided into eight groups (n = 3), and they were injected intravenously with different nanoparticles. At 36 h postinjection, the tumors were collected for cryosection and stained by DCFH-DA for CLSM observation. For in vivo GSH assessment, the tumor samples derived from each group were frozen with liquid nitrogen for powder grinding. The quantification of GSH in the supernatants was assessed by GSH and GSSG Assay Kit, according to the manufacturer's instruction.

Gating strategies for immune cells

In the gating strategy used to identify leukocytes, a nucleated cell gate based on FSC-A and SSC-A was set to remove debris for single cell analysis. FSC-A and FSC-H were used to exclude doublets and cell aggregates. Next, the T leukocytes were identified on the basis of CD3 expression. T cells were further investigated for the expression of CD4 and CD8, which resulted in CD4+T and CD8+T cell populations. On the basis of the co-expression of CD11b and Gr-1, a heterogeneous group of myeloid cells, MDSCs presented immunosuppressive ability. After cell doublets, clumps, and debris were eliminated, the MDSCs were identified on the basis of CD11b and Gr-1 co-expression.

A lymphocyte gate and a combined expression of CD4 and Foxp3 were used to identify Tregs.

F4/80 is a widely used marker for macrophages, and CD206, the mannose receptor expressed on macrophages, has been used as a marker for M2 macrophage polarization. We excluded debris, cell clumps, and dead cells to characterize the M2 TAM cells in tumor tissues. F4/80 and CD206 expression patterns were used to identify M2 macrophage cells.

Table S1. The full name of each groups' abbreviation

Abbreviation	Full name
CON	PBS
RT	Cu-GA
siVEGF	siVEGF
BRT	Cu-GA- siVEGF
HL	Hypoxia-triggerable liposomal
HLB	Hypoxia-triggerable liposomal loaded with BMS
HLBRT	Hypoxia-triggerable liposomal loaded with BRT
HLBBRT	Hypoxia-triggerable liposomal loaded with BMS and BTR

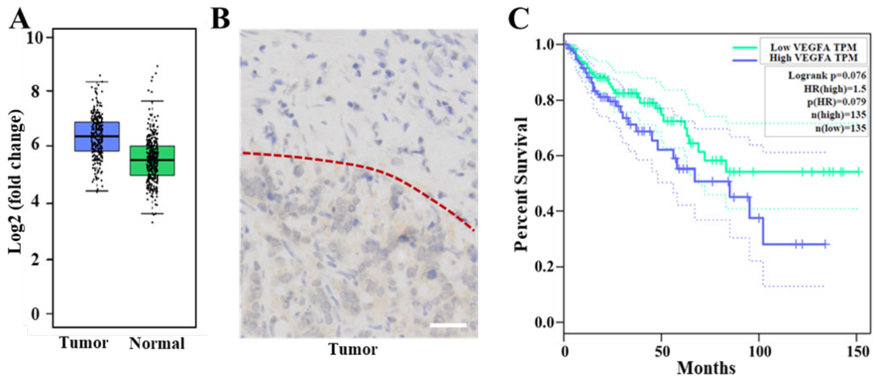

Figure S1. (A) Bioinformatical analysis of VEGF gene expression level in COAD patients based on TCGA database. (B) Immunohistochemical analysis of VEGF in tumor tissues and adjacent normal tissues. Scale bar, 25 µm. (C) The overall survival of COAD patients with high or low VEGF expression.

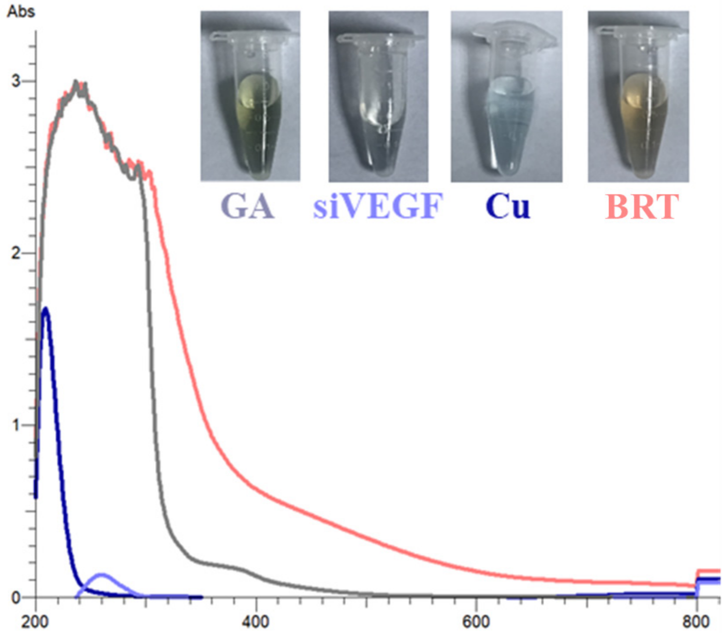

Figure S2. UV-vis spectrum of GA (gray), siVEGF (purple), Cu (blue) and BRT (red).

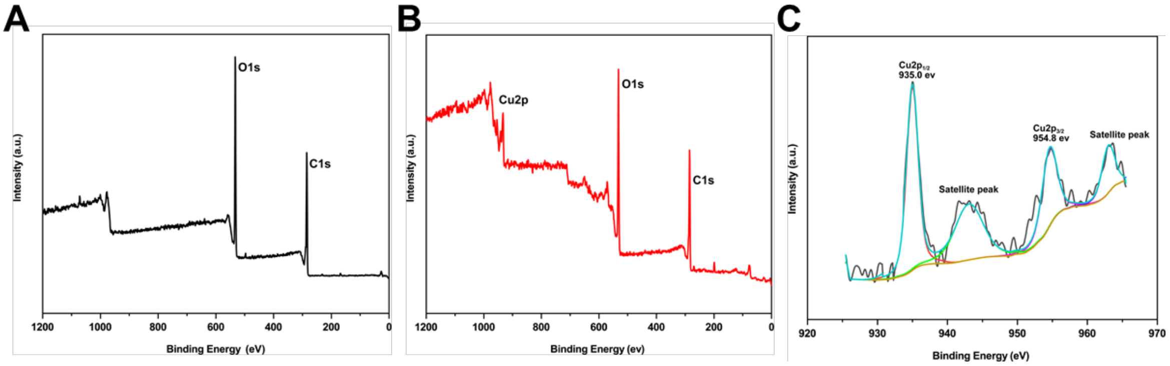

Figure S3. X-ray photoelectron spectra (XPS) of (A) GA, (B) Cu-GA and (C) Cu 2p oribit for GA-Cu nanoparticle.

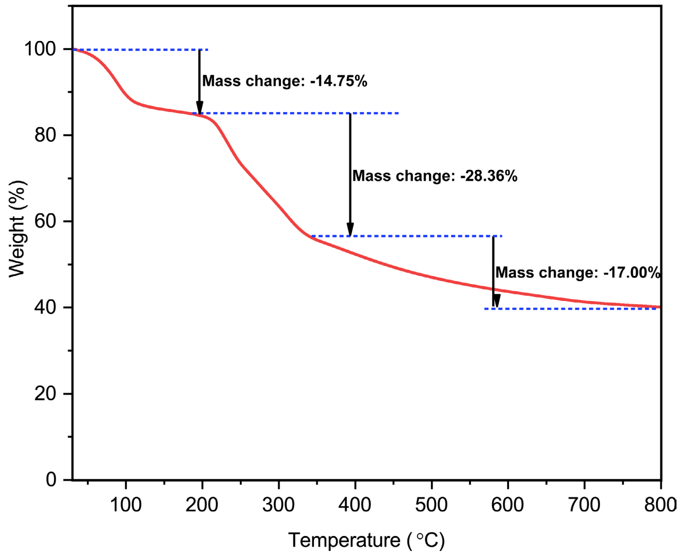

Figure S4. Thermogravimetric analysis curves of Cu-GA.

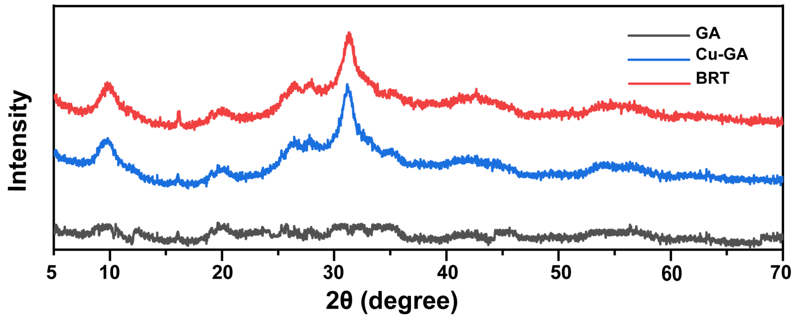

Figure S5. XRD patterns of GA, Cu-GA and BRT.

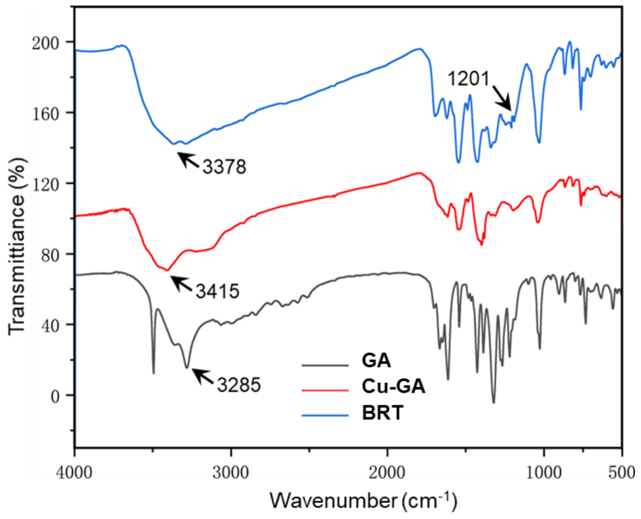

Figure S6. FT-IR spectra of GA, Cu-GA and BRT.

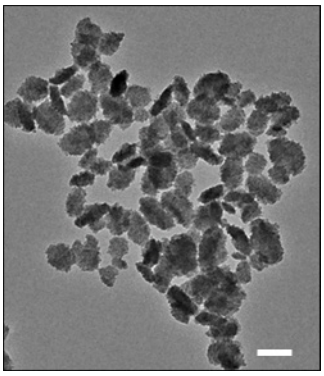

Figure S7. TEM images of BRT (scale bar = 100 nm).

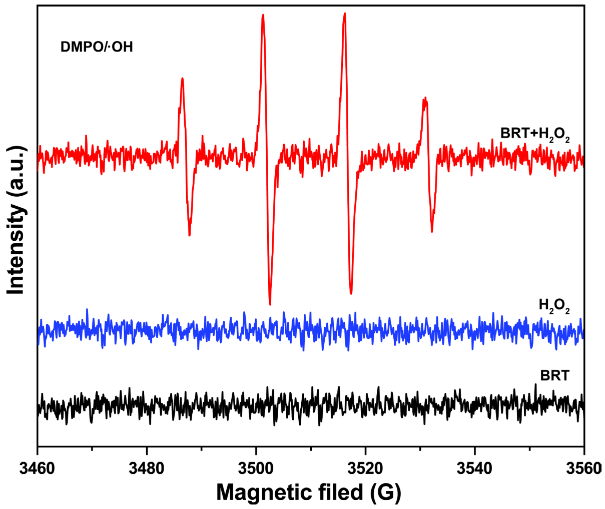

Figure S8. ESR spectra of different agents with DMPO as the radical trap.

A

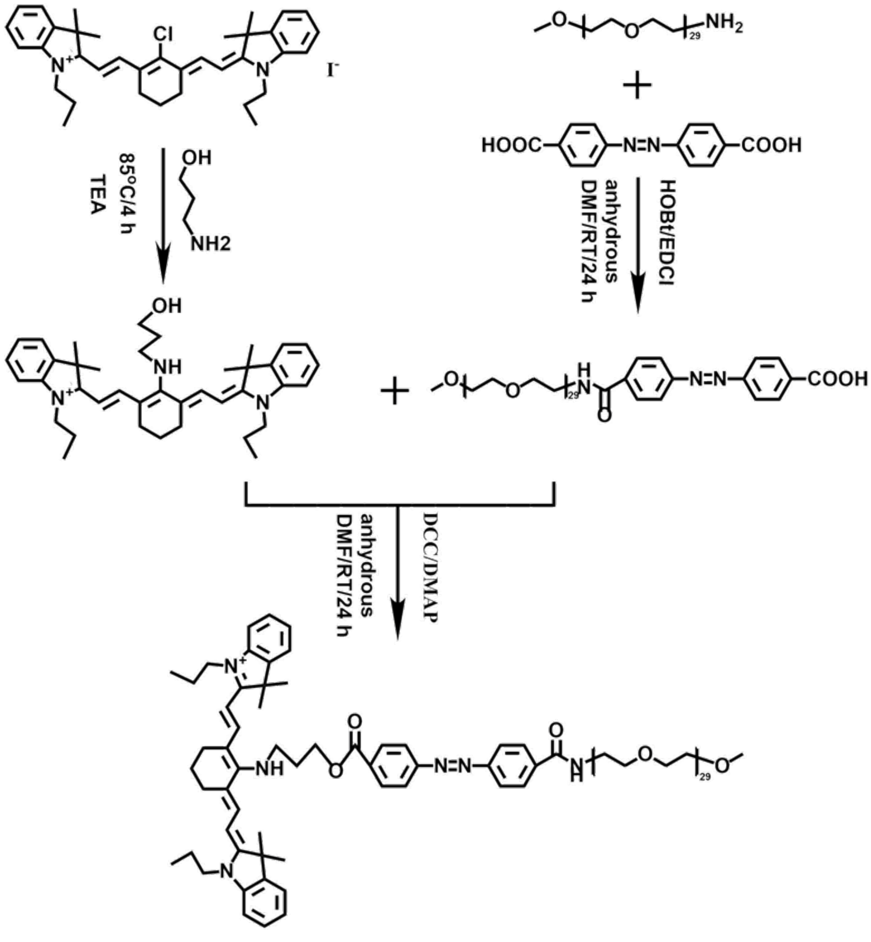

Cl
I⁻
85°C/4 h
TEA
OH
NH2
NH2
HOOC
N=N
COOH
HOBt/EDCI
anhydrous
DMF/RT/24 h
29
DCC/DMAP

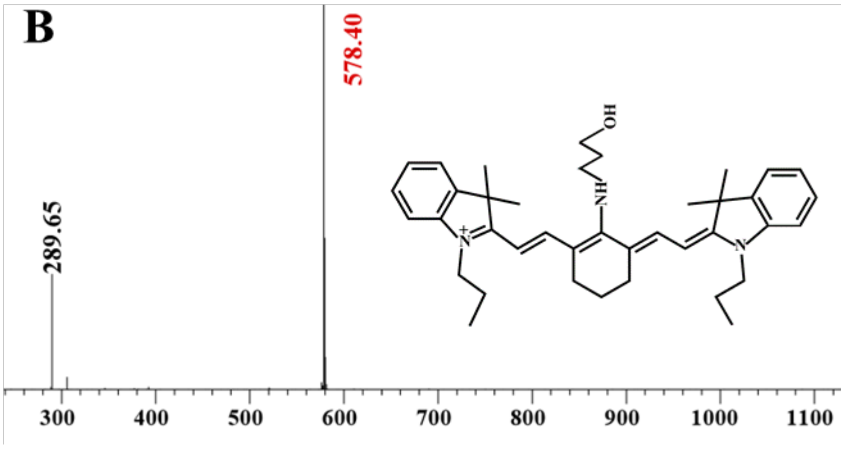
B
578.40
289.65
OH
NH
N+
N
300
400
500
600
700
800
900
1000
1100

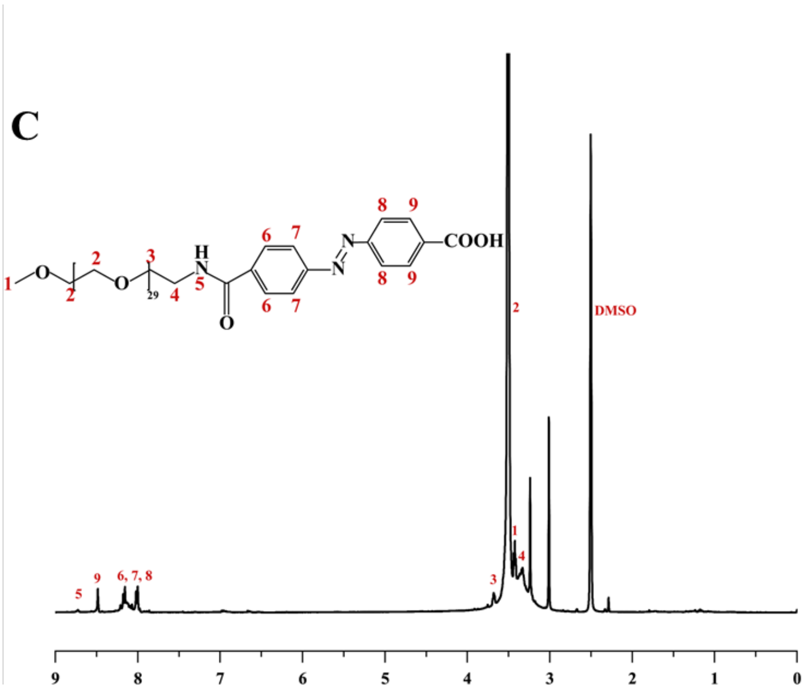
C
COOH
H
N
O
29
DMSO
5
9
6, 7, 8
3
1
4
2
9
8
7
6
5
4
3
2
1
0

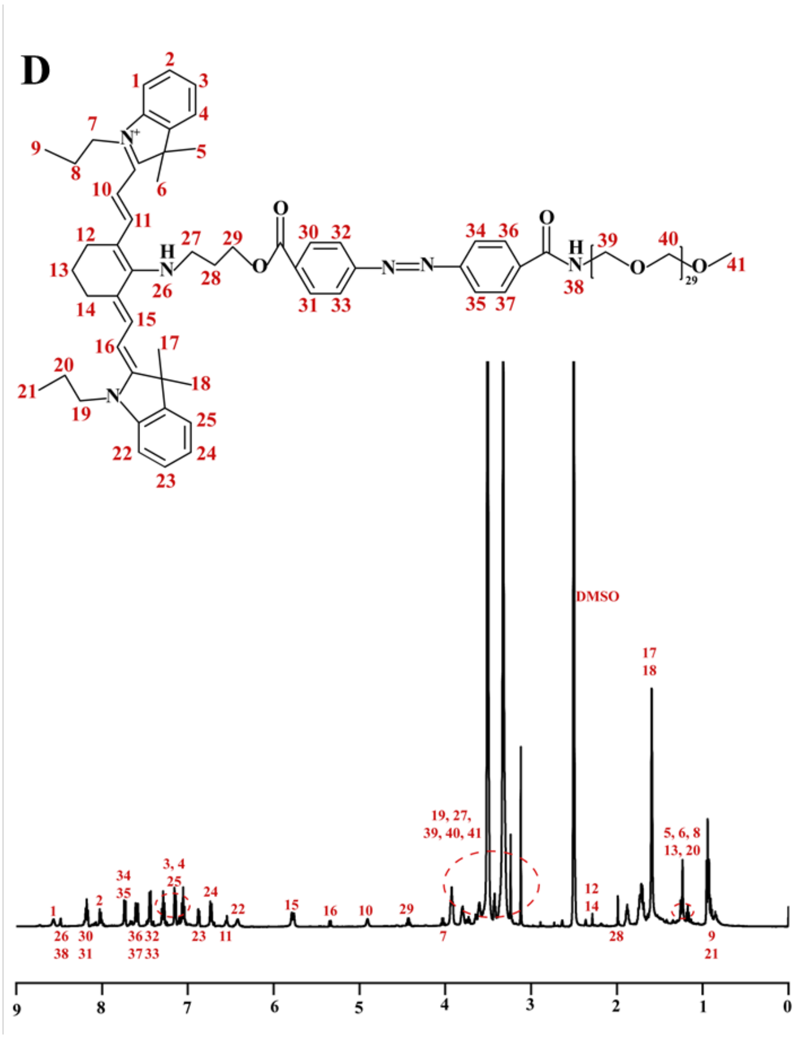

Figure S9. (A) Synthetic of PEG-AZO-IR. (B) ESI-MS spectra of IR-OH. (C) ^{1}H-NMR spectra of PEG-AZO and PEG-AZO-IR (D).

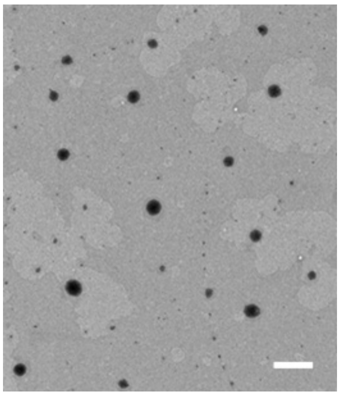

Figure S10. TEM images of HLBBRT NPs (scale bar = 500 nm).

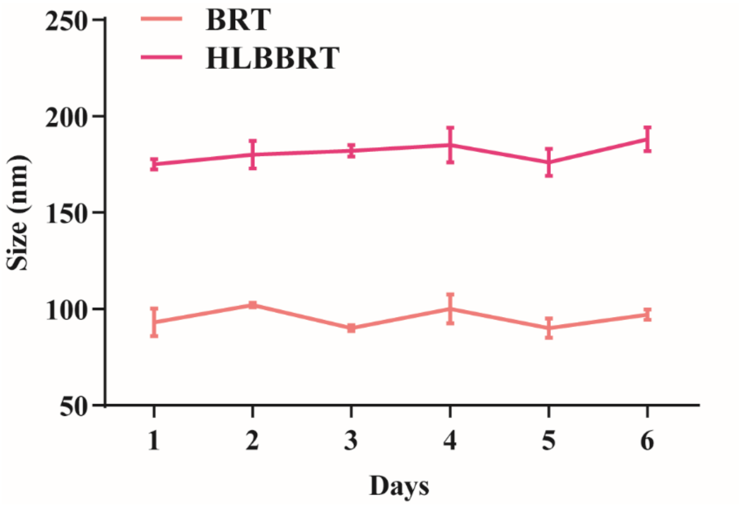

Figure S11. The stability of particle size distribution of BRT and HLBBRT in 6 days.

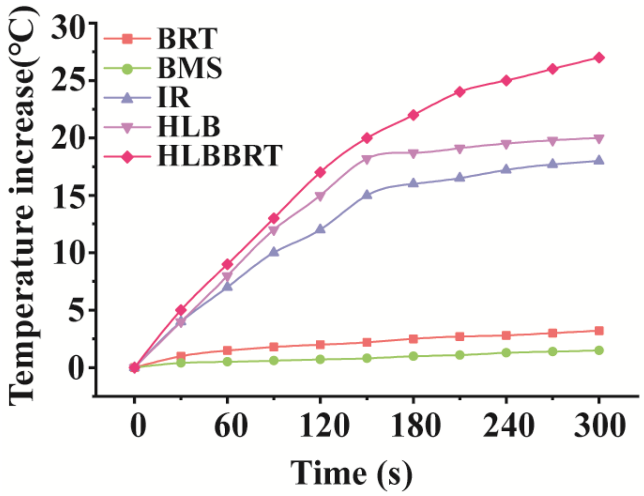

Figure S12. Temperature-increase curves of solutions containing each formulation under irradiation of 808 nm laser (1 W cm^{-2}) in 5 min.

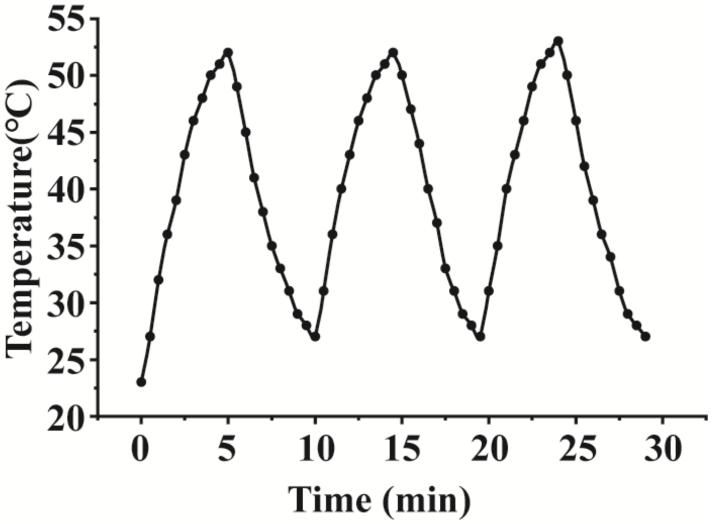

Figure S13. Temperature changing curves of the HLBBRT solutions for three on/off cycles at the 808 nm laser irradiation (1 W cm^{-2}).

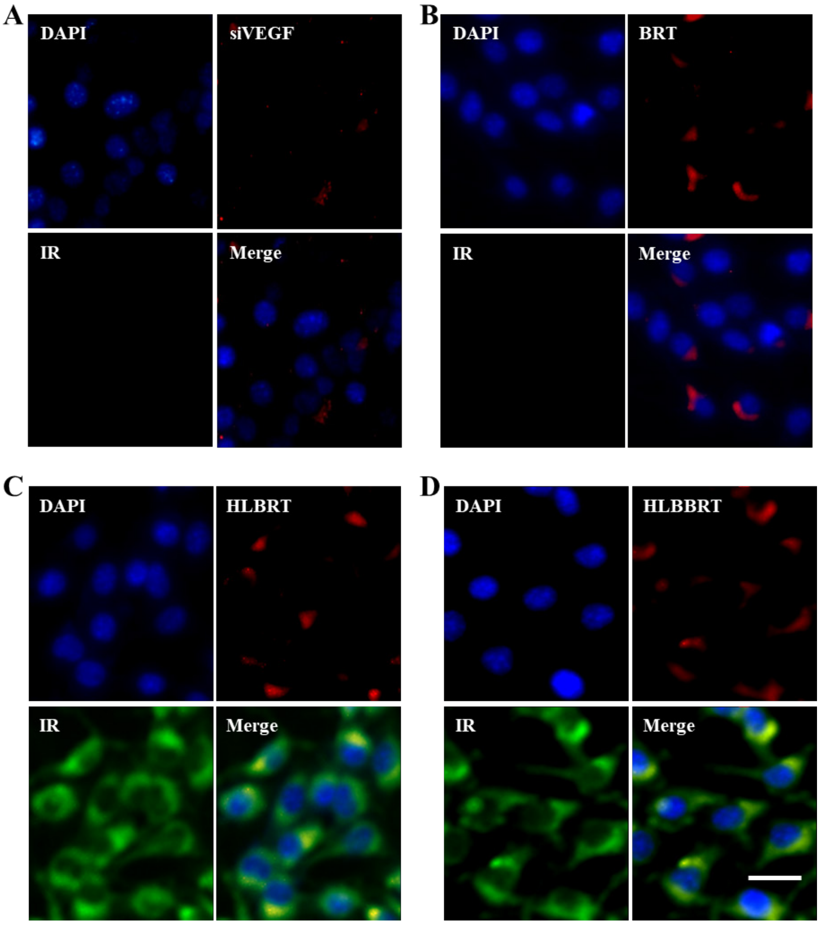

Figure S14. CLSM images of cellular uptake in CT26 cells after 6 h incubation with different formulations under hypoxic conditions. (A) Free siVEGF and IR; (B) BRT and free IR; (C) HLBRT NPs; (D) HLBBRT NPs. Blue, red, and green colors represent DAPI, FAM-labeled siVEGF, and IR fluorescence, respectively (Scale bar, 5 μm).

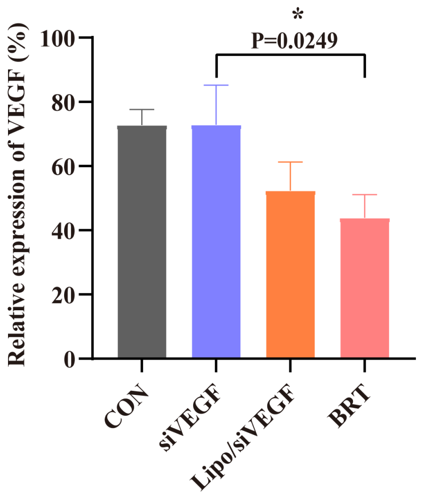

Figure S15. The relative VEGF expressions were normalized with the β-actin expression. Indicated error bar represents the mean ± S.D. (n=3).

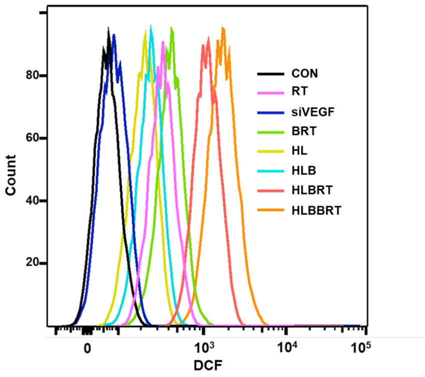

Figure S16. Flow cytometry of intracellular total ROS radicals by using DCFH-DA as a probe.

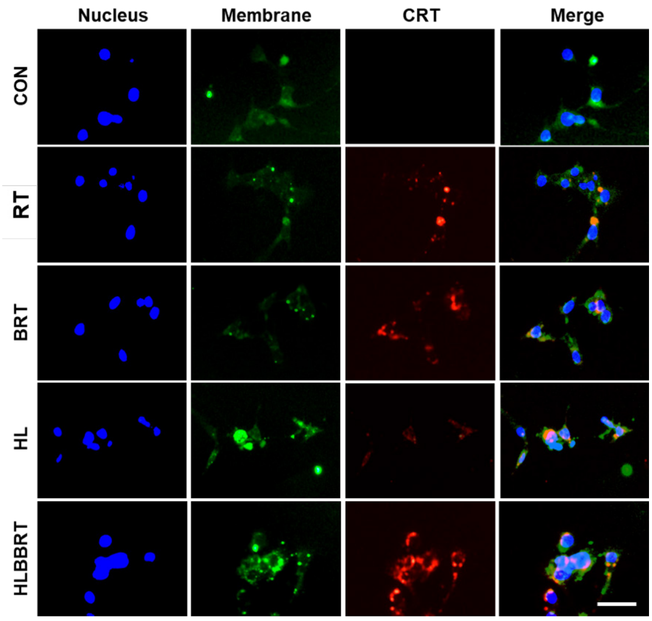

Figure S17. CLSM images detection of CRT exposure on the surface of CT26 cells (scale bar: 25 μm)

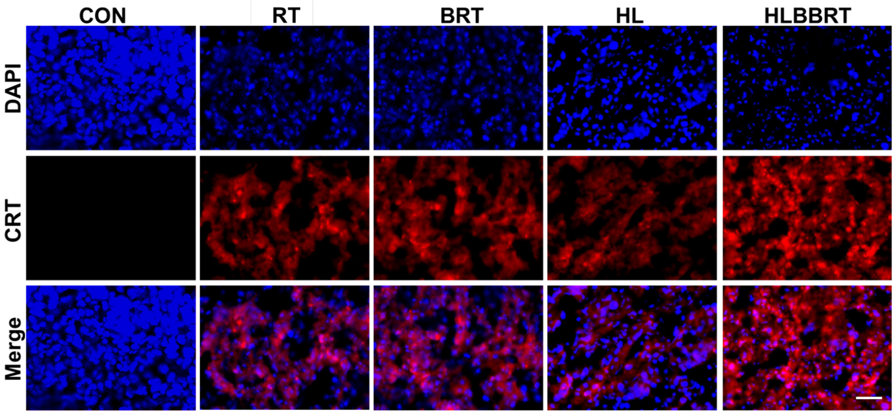

Figure S18. CRT expression of tumor sections (scale bar: 50 μm).

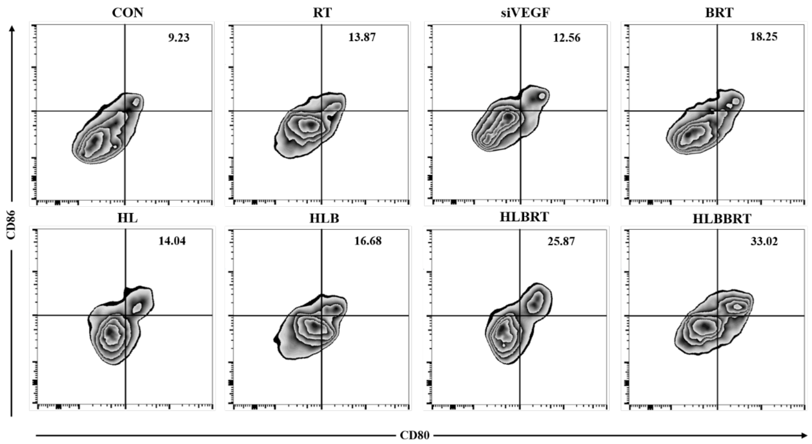

Figure S19. Flow cytometry results of the DCs maturation induced by various nanoparticles *in vitro*.

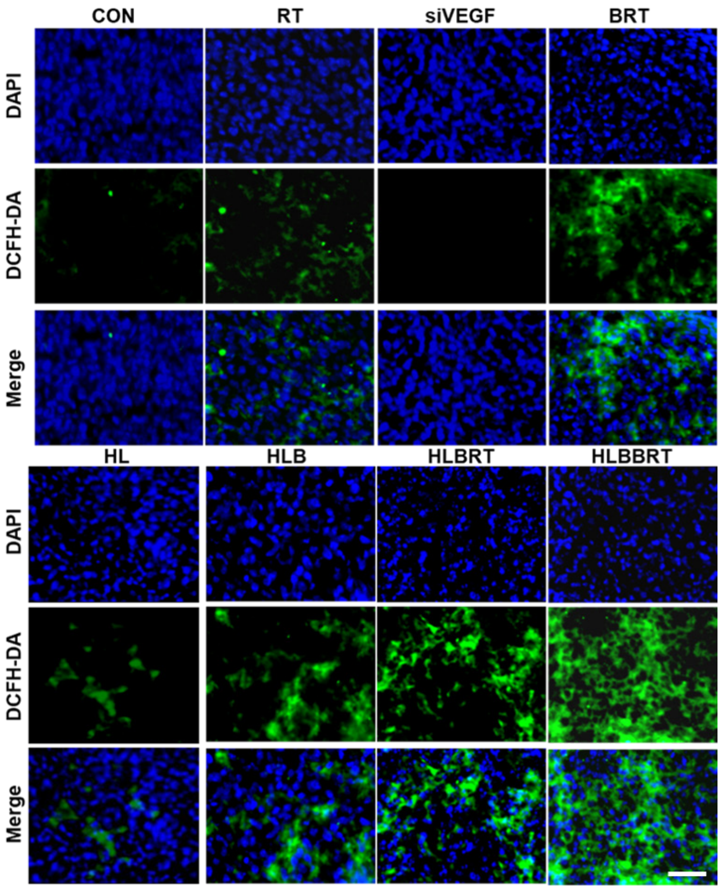

Figure S20. Images of tumor tissues stained with DCFH-DA (scale bar: 50 μm).

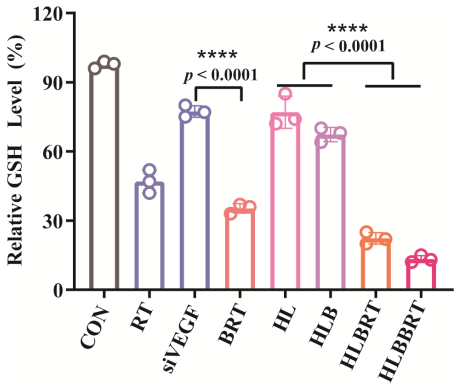

Figure S21. GSH level analysis in tumor tissues.

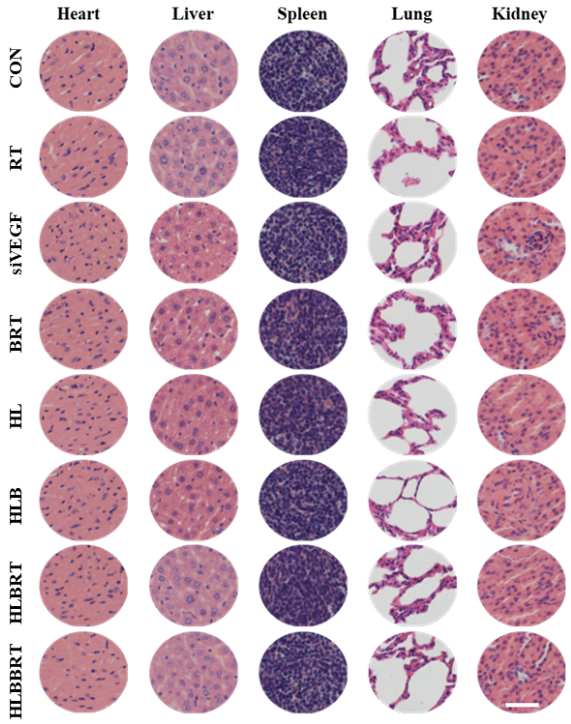

Figure S22. H&E staining analysis of main organs after various treatments for 21 days. The scale bar is 25 μm.

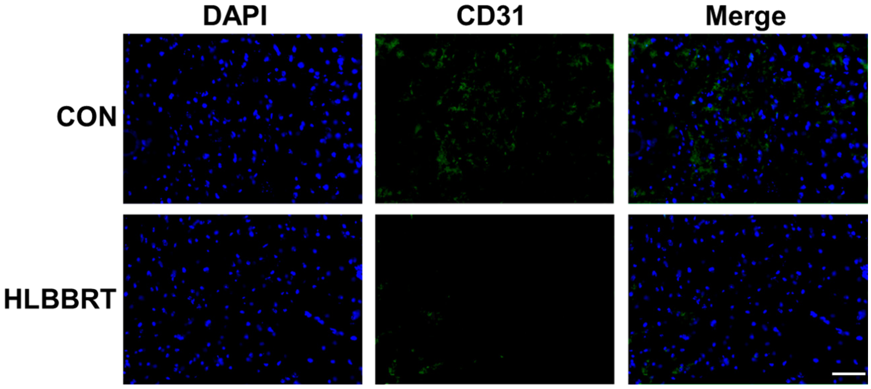

Figure S23. The CLSM images of CD31 staining in tumor sections (scale bar: 50 μm).

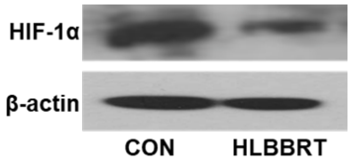

Figure S24. Western-blot analysis of HIF-1α expression in tumor tissue of CON and HLBBRT NPs treated groups.

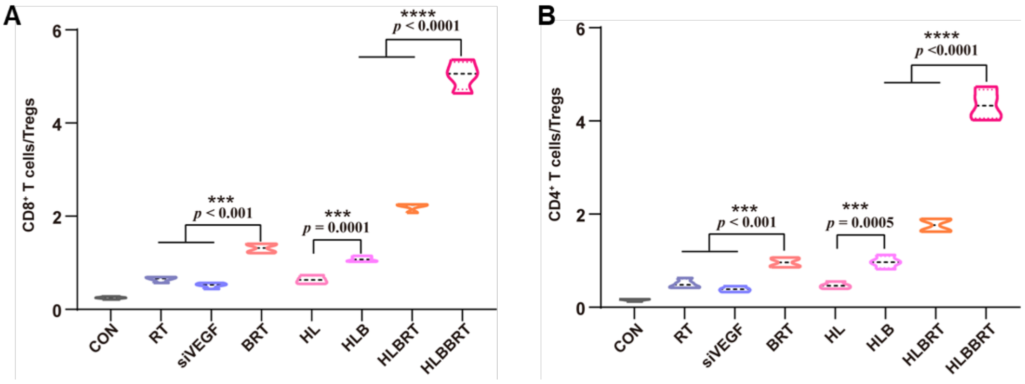

Figure S25. Ratios of (A) CD8+ T cells to Tregs and (B) CD4+ T cells to Tregs in tumors of different groups (n = 4).

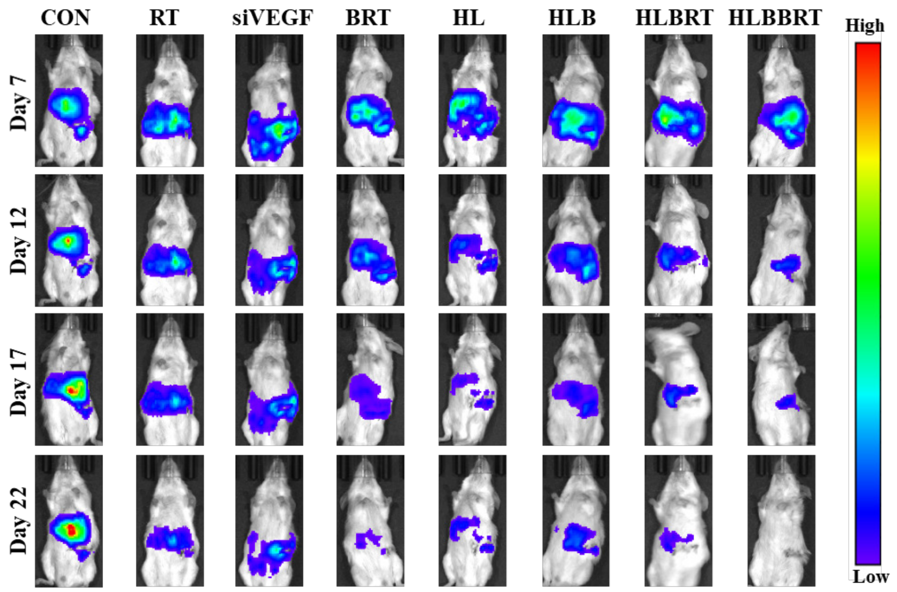

Figure S26. *In vivo* bioluminescence images tracking the growth of CRC liver metastasis in the mice after different treatments.

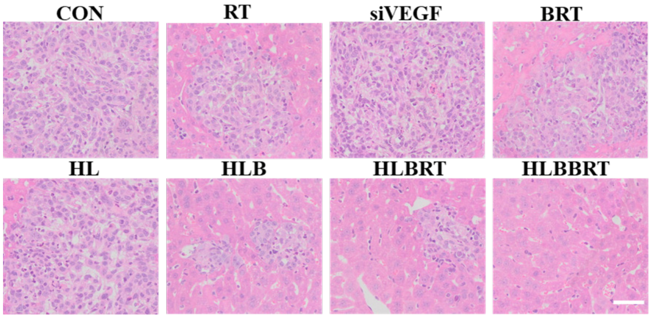

Figure S27. H&E (Scale bar, 50 μm) analysis for tumor sections of mice at the end of each treatment.

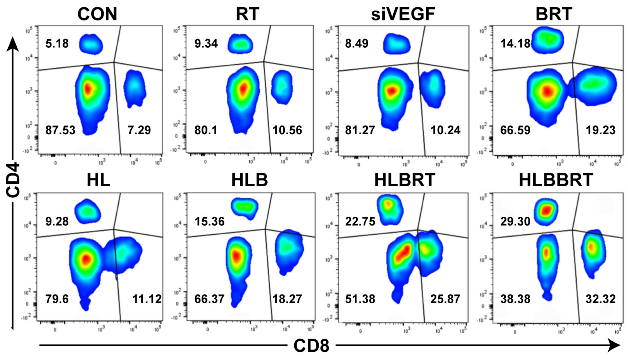

Figure S28. Representative flow cytometry analysis of the $CD4^+$ and $CD8^+$ T cells.
